# Micro- and Nanoplastics and Human Health: Role of Food Nutrients Targeting *Nfe2l2* Gene in Diabetes

**DOI:** 10.3390/nu18040600

**Published:** 2026-02-11

**Authors:** Maria Concetta Scuto, Cinzia Lombardo, Nicolò Musso, Paolo Giuseppe Bonacci, Gabriella Lupo, Carmelina Daniela Anfuso, Angela Trovato Salinaro

**Affiliations:** 1 Department of Medicine and Surgery, Kore University of Enna, 94100 Enna, Italy; mariaconcetta.scuto@unikore.it (M.C.S.); nicolo.musso@unikore.it (N.M.); 2 Department of Biomedical and Biotechnological Sciences, School of Medicine, University of Catania, 95123 Catania, Italy; cinzia.lombardo@unict.it (C.L.); paolo.bonacci@phd.unict.it (P.G.B.); gabriella.lupo@unict.it (G.L.); daniela.anfuso@unict.it (C.D.A.)

**Keywords:** microplastics, nanoplastics, food nutrients, *Nfe2l2* gene, epigenetic modifications, pyroptosis, inflammation, diabetes, nephropathy, neuropathy

## Abstract

A new category of polyphenolic compounds, like flavonoids, phenolic acids, phenylpropanoids, terpenoids, and others, referred to as food nutrients, may counteract the harmful effects of micro- and nanoplastics (MNPs) by enhancing cellular stress resilience response and overall human health. These compounds found in functional food help mitigate the cellular damage, inflammation, and oxidative stress caused by MNP exposure, which can contribute to pathological conditions, including diabetes. Importantly, specific food nutrients are able to activate, at the minimum dose, the nuclear factor erythroid-derived 2-like 2 (Nrf2) to prevent or block MNP-induced damage. The *Nfe2l2* gene encodes the Nrf2 transcription factor, acting as a master regulator of redox homeostasis by inducing antioxidant response element (ARE)-driven resilience genes, which in turn, promote the expression of detoxification enzymes like heme oxygenase-1 (HO-1), NAD(P)H: quinone oxidoreductase 1 (NQO1), and glutathione S-transferase (GST) to scavenge reactive oxygen species (ROS) and shield cells from environmental damage and toxicity. Deregulation of the *Nfe2l2* gene due to the accumulation of MNP pollutants may exacerbate the inflammatory conditions associated with diabetes and its chronic complications by rendering cells more sensitive to oxidative stress, apoptosis, and pyroptosis. Furthermore, epigenetic modifications influence gene regulation; chromatin remodeling directly impacts DNA accessibility, allowing or limiting transcription factor access to regulate gene expression. This mechanism may also play a pivotal role in the progression of oxidative stress-related diseases, as it modulates the Nrf2 pathway and the expression levels of its target genes. In contrast to the current literature, which has only addressed the pathological mechanisms induced by MNPs, this research explores, for the first time, how food nutrients interacting with the *Nfe2l2* gene can combat or reverse the toxic effects of MNPs in cells, tissues, and organs. The goal is to improve health by attenuating MNP toxicity, which is influenced by individual genetic variations and cellular stress resilience.

## 1. Introduction

Micro- and nanoplastics (MNPs) represent a growing environmental concern due to their potential health impacts. Human exposure to these small plastic particles occurs through the ingestion of contaminated beverages and foods and the inhalation of particles from other plastics and textiles [1]. Microplastics (MPs) are usually defined as plastic particles less than 5 μm in diameter [2]. After additional breakdown or degradation, MPs become nanoplastics (NPs), particles smaller than 1 μm in diameter [3]. Several studies have shown that polyethylene terephthalate (PET), polypropylene (PP), polyethylene (PE), polyvinyl chloride (PVC), polycarbonate (PC), polyamide (PA), and polystyrene (PS) are the most common plastic particles present in food [1]. By 2025, global plastic production is expected to reach approximately 400 million tons. Furthermore, the projection in the study by Cincinelli et al. of a cumulative production of 33 billion tons by 2050 represents a worrying scenario [4,5]. Some studies suggest that MNPs can affect human health by activating several mechanisms, such as oxidative stress, inflammation, cellular barrier disruption, and pyroptosis [1]. In this context, diabetes mellitus (DM) is a chronic metabolic disorder characterized by persistent blood glucose levels that is caused by insulin resistance (IR) and inadequate compensatory secretion of insulin. Diabetes is a major public health concern, with type-2 diabetes mellitus (T2DM) accounting for over 90% of cases and type-1 diabetes affecting a smaller but increasing number of young people [6]. Recent research has suggested that MPs can cause abnormal glucose and lipid metabolism [7], directly linking plastic exposure to the onset of diabetes and its complications, such as neuropathy and nephropathy. In diabetes, elevated blood glucose levels (hyperglycemia) result from a failure in the insulin–glucose regulation loop, involving deficient insulin secretion from pancreatic islet β-cells or reduced insulin sensitivity in tissues, such as the liver, adipose tissue, and muscle. Chronic hyperglycemia and IR are key features of diabetes that promote the formation of advanced glycation end products (AGEs) through chronic hyperglycemia. AGEs can cause cellular damage and inflammation, leading to complications such as diabetic nephropathy and diabetic neuropathy by creating irreversible crosslinks in proteins and triggering a toxic cascade of harmful cellular responses. Moreover, IR is involved in impaired insulin signaling and molecular pathways, including mitogen-activated protein kinases (MAPK), extracellular signal-regulated kinases 1 and 2 (ERK1/2), and c-Jun *N*-terminal kinase (JNK1-3), which can disrupt normal insulin signaling. These pathways are also interconnected with others, such as the Nrf2, p38, glycogen synthase kinase 3 β (GSK3β), mammalian target of rapamycin (mTOR), phosphoinositide 3-kinases (PI3Ks), and phosphorylation of protein kinase B (AKT). Dysregulation in these systems contributes to the development of T2DM [8]. Noteworthy is the growing interest in the interrelationships between peripheral neuropathy and nephropathy in patients with T2DM [9,10]. Pathophysiological mechanisms associated with IR and the onset of diabetes and its complications include increased oxidative stress, insulin signaling disorder, AGEs, pyroptosis, mitochondrial dysfunction, and neuroinflammation [11]. This evidence is consistent with research indicating that exposure to PS-MPs, particularly those with a diameter of 1 μm or less, can induce insulin resistance (IR) [12] and worsen glucose tolerance [13] in mice through metabolic disturbances of the gut–liver axis. Moreover, exposure to amino-modified polystyrene nanoplastics (PS-NPs-NH2) can inhibit the phosphorylation of AKT and FoxO1, disrupting glucose metabolism and leading to the development of T2DM-like lesions [14]. Similarly, PS-MPs (0.5 µm) in db/db mice worsened kidney damage by activating the NLRP3/caspase-1 pro-inflammatory cascade, and triggering the transforming growth factor-β1 (TGF-β1)/Smad signaling pathway, leading to fibrosis and tissue damage [15]. Recent research has suggested that MPs can cause abnormal glucose and lipid metabolism [7], as they indicate that exposure to PS-MPs, particularly those with a diameter of 1 μm or less, can induce IR [12] and worsen glucose tolerance [13] in mice via gut–liver axis metabolic disturbances. Moreover, exposure to amino-modified polystyrene nanoplastics (PS-NPs-NH2) can inhibit the phosphorylation of AKT and FoxO1, which disrupts glucose metabolism, leading to elevated blood glucose levels and the development of T2DM-like lesions [14]. Similarly, PS-MPs (0.5 µm) in db/db mice worsened kidney damage by increasing oxidative stress, activating the NLRP3/caspase-1 inflammatory pathway, and triggering the TGF-β1/Smad signaling pathway, leading to tissue damage and fibrosis [15]. Oxidative stress, caused by an imbalance between free radicals and intracellular antioxidants, damages cells by harming proteins, lipids, and DNA. This process is highly regulated by Nrf2, encoded by the *Nfe2l2* gene, which is the master regulator of phase II detoxification enzymes. Normally, Nrf2 is complexed with Kelch-like erythroid cell-derived protein with CNC homology (ECH)-associated protein 1 (Keap1) in the cytoplasm to mediate its ubiquitination and proteasome degradation. Under stress, Keap1-Nrf2 interaction is disrupted, allowing Nrf2 to stabilize, translocate to the nucleus, and bind to phase 2 of the antioxidant response element (ARE). This cellular redox mechanism activates gene transcription for stress resilience proteins and enzymes, including heat shock protein 70 (Hsp70), heme oxygenase-1 (HO-1), sirtuin-1 (Sirt1), the thioredoxin (Trx)/thioredoxin reductase system, NADPH quinone oxidoreductase 1 (NQO1), γ-glutamylcysteine synthetase (γ-GCs), superoxide dismutase (SOD), catalase (CAT), glutathione (GSH), glutathione peroxidase (GPx), and forkhead box O3 (FOXO3) to maintain metabolic homeostasis and restore stress adaptation against MNP-induced damage and the potential onset of several chronic disorders [16,17,18]. In this context, food nutrients can activate cellular stress resilience responses, offering a novel strategy for personalized nutrition to combat MNP-induced oxidative stress and inflammation occurring in T2DM, diabetic nephropathy, and diabetic neuropathy. This approach focuses on using specific nutrients like phenols, flavonoids, tannins, alkaloids, and triterpenoids, widely distributed in various parts of plants, to activate the *Nfe2l2* gene and trigger the body’s endogenous stress-resilient defense mechanisms and pathways [18,19,20]. The research explores, for the first time, nutrient–gene interactions, where specific food nutrients activate the antioxidant *Nfe2l2* gene and stress resilience proteins to counteract oxidative stress, pyroptosis, and epigenetic changes caused by MNPs, ultimately preserving metabolic health. Identifying how the *Nfe2l2* gene and nutrients interact holds immense promise for the development of personalized dietary recommendations based on individual genetic and epigenetic profiles to inhibit or reverse MNP-induced cell damage at the transcriptional, post-transcriptional, and post-translational levels. Moreover, the review reports advanced 3D models for studying novel nutritional interventions, with the ultimate goal of identifying novel antioxidant biomarkers of stress resilience response activated by specific food nutrients to personalize nutritional counseling and support physicians and nutritionists in developing highly targeted recommendations for the management of diabetes and its complications, based on individual patients’ genetic, environmental, and lifestyle factors, as suggested by the concept of precision nutrition.

## 2. Narrative Review Search Strategy and Selection Criteria for Nutrients

We performed comprehensive searches of the literature in PubMed, Web of Science, and Scopus (2014–January 2026). Search terms combined “food nutrients”, “polyphenols”, “Nrf2”, “oxidative stress”, “pyroptosis”, “genetic variations”, “epigenetic modifications”, “inflammation”, “diabetes”, “diabetic complications”, “microplastics”, and “nanoplastics”. Both preclinical and clinical studies were included.

The inclusion criteria were (i) original studies or review and systemic review/meta-analysis; (ii) in vitro and in vivo studies and human clinical trials addressing personalized nutritional interventions relevant to diabetes and its complications; (iii) microplastics and nanoplastics and human health outcomes; (iv) data on molecular mechanisms and clinical outcomes; and (vi) innovative in vitro models to study the interaction between food nutrients and diabetic pathology.

The exclusion criteria were (i) studies without molecular mechanisms or microplastic and nanoplastic endpoints and (ii) case reports or interventions unrelated to a personalized nutritional approach.

The selection of nutrients was guided by the following criteria: (i) high ability to cross the gut barrier; (ii) documented specific efficacy against diabetic complications, such as nephropathy and/or neuropathy; and (iii) recent studies (2020–2024) that specifically link these compounds to the cellular stress response through modulation of the Nrf2 pathway.

The compounds were chosen for their properties that exert local antioxidant and anti-inflammatory protection at the intestinal barrier, which is the primary site of interaction when MNPs are ingested.

## 3. MNPs and the Potential Risk of Diabetes and Its Complications

Environmental pollution by MNPs poses significant human health risks, as reported by studies showing accumulation in vital organs, including the liver, kidneys, and pancreas [21]. These particles trigger metabolic dysfunction, oxidative stress, and inflammation, severely compromising glucose homeostasis and lipid metabolism by affecting multiple pathways. Indeed, NPs affected the PI3K/Akt signaling pathway, disrupting glucose metabolism. The increased phosphorylation of insulin receptor substrate-1 inhibits the PI3K/Akt pathway, resulting in IR and high plasma glucose in diabetic models [21]. Based on the findings, combined exposure to MPs induces IR and impaired glucose tolerance in mice via the Nrf2/NF-κB signaling pathway by increasing phosphorylated p-NF-κB protein levels and reducing Nrf2 mRNA and HO-1 protein levels. Similarly, mice exposed to PS-NPs modified by different functional groups at a dose of 5 mg/kg/day for nine weeks exhibited elevated fasting blood glucose levels, glucose intolerance, and IR via the inhibition of the P-AKT/P-FoxO1 pathway [14]. These conditions were more severe in diabetic mice. Blood glucose, glucose intolerance, and IR were increased after oral exposure to 30 mg/kg/day of polystyrene NPs for 8 weeks through a reduction in AKT/GSK3β phosphorylation, exacerbating T2DM in experimental mouse models [13]. Oral administration of 0.5 µm PS-MPs markedly increased the oxidative redox state and inflammation, thereby aggravating kidney injury and renal fibrosis by activating NLRP3/Caspase-1/TGF-β1/Smad signaling pathways in db/db mice [15]. Network toxicology analysis integrated with machine-learning models demonstrated that chronic exposure to polyethylene terephthalate microplastics (PET-MP) promoted nephrotoxicity targeting central differentially expressed genes, such as PIK3R1, PIK3CA, PIK3CB, NR3C2, CASP3, and GRB2, involved in exacerbating DN progression [22]. A recent comparative study suggested that 100 nm-PS-MPs exposure is more toxic than 5 µm-PS-MPs. In fact, small particle size exposure of 100 nm-PS-MPs significantly increased liver inflammation (TNF-α and IL-1β), altered intestinal microbial composition, and intensified hepatic lipid disorders in diabetic mice [23]. Additionally, MP exposure of 0.5 μm for 3 months induced oxidative stress and hepatic gluconeogenesis by altering the PP2A/AMPK/HNF4A signaling pathway [24]. Plastic mixtures containing MPs and NPs significantly increased adipogenic differentiation (*PPARγ*) and synthesis (*FASN* and *FABP*), inflammatory cytokines (*TNF-α* and *IL-6*), and gluconeogenesis (*PCK1* and *G6Pase*). Conversely, energy and fat metabolism (*AMPKα* and *adiponectin*), insulin production (*INSα*), signaling pathway (*IRS1*, *AKT*, and *GLUT2*), and anti-inflammatory cytokines (*IL-10* and *IL-4*) were suppressed in zebrafish [25]. Short-term PS exposure to PS-MPs of about 3 μm aggravated the intestinal damage correlated with metabolic changes, in particular cholesterol sulfate, ascorbic acid 2-sulfate, and trigonelline and valerylcarnitine that were downregulated in diabetic rats [26]. Although a dose of 5 mg/kg/day in mouse models does not translate linearly to humans, this dose is equivalent to approximately 0.4 mg/kg in humans, a value much closer to potential exposure levels in cases of significant environmental or dietary contamination [27]. Unlike laboratory studies, which are usually conducted in the short term, i.e., weeks, human exposure lasts for decades and can therefore be defined as chronic bioaccumulation. High short-term doses serve to simulate the tissue accumulation that occurs in humans [28,29]. Humans are exposed via water and through a combination of ingestion, inhalation, and dermal contact. Recent estimates suggest that the average individual may ingest between 0.1 and 5 g of MPs per week [27]. While subacute exposures are used in animal models to observe short-term molecular mechanisms, such as Nrf2 activation, human exposure is chronic and cumulative. Nanosized particles, thanks to their small size, can cross biological barriers and accumulate in the pancreas, where their local concentration can reach critical levels over time, promoting insulin resistance [12]. Overall, these experimental findings suggest that exposure to MNPs leads to elevated blood glucose levels, IR, and ultimately metabolic dysfunction. However, there are no direct, long-term human clinical studies confirming that MNPs cause diabetes. Further studies are essential to comprehensively understand the underlying molecular mechanisms of MNP toxicity in diabetic patients and to develop personalized strategies for preventing these risks. Key research priorities include identifying long-term, low-dose exposure effects and standardizing metrics for toxicity.

## 4. MNPs Induce NLRP3 Inflammasome and Pyroptosis in Diabetic Complications

**Figure 1:**
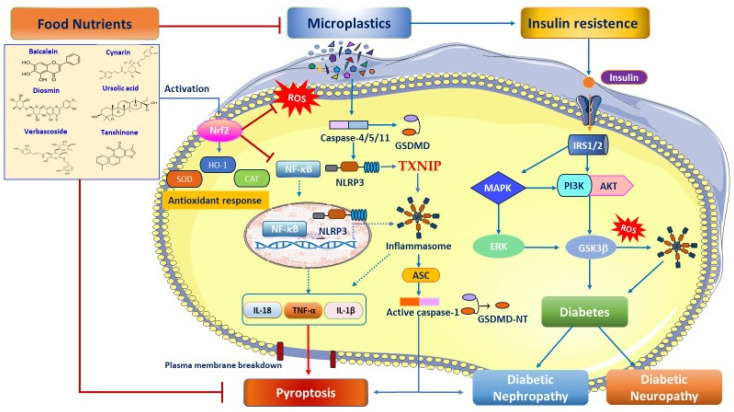
Schematic diagram of nutritional medicine targeting Nrf2 signaling pathway to inhibit microplastic-induced NLRP3 inflammasome activation and pyroptosis in diabetes and related complications. ROS—reactive oxygen species; Nrf2—nuclear factor erythroid 2-related factor 2; NF-κB—nuclear factor kappa-light-chain enhancer of activated B cells; HO-1—heme oxygenase-1; SOD—superoxide dismutase; CAT—catalase; NLRP3—NLR family pyrin domain containing 3; GSDMD—gasdermin D; TXNIP—thioredoxin-interacting protein; IL-18—interleukin-18; TNF-α—tumor necrosis factor alpha; IL-1β—interleukin-1 beta; ASC—apoptosis-associated speck-like protein containing a CARD; GSDMD-NT—gasdermin D *N*-terminal; IRS1—insulin receptor substrate 1; IRS2—insulin receptor substrate 2; MAPK—mitogen-activated protein kinase; ERK—extracellular signal-regulated kinase; PI3K—phosphoinositide 3-kinase; Akt—protein kinase B; GSK3β—glycogen synthase kinase-3 beta.

Pyroptosis is a highly inflammatory form of programmed cell death, mediated by gasdermin family proteins, especially gasdermin D (GSDMD). This protein forms pores in the cell membrane, leading to cell swelling, rupture, and the release of potent pro-inflammatory cytokines like tumor necrosis factor-α (TNF-α), interleukin-1β (IL-1β), and IL-18, crucial for innate immunity but also implicated in various chronic inflammatory diseases, including tumors, nerve injury, and metabolic disorders, when their delicate homeostasis is disrupted [30]. Mechanistically, pyroptosis is initiated by inflammasome-activated caspases (caspase-1, 4, 5, 11) or apoptotic caspases (caspase-3, 8). These caspases proteolytically cleave GSDMD into an *N*-terminal pore-forming domain (PFD) and the *C*-terminal repressor domain (RD). Free PFD oligomers move to the plasma membrane and bind to phosphoinositides, creating membrane pores (diameter 10–20 nm). This causes osmotic swelling, membrane rupture, and the release of inflammatory cytokines (IL-1β and IL-18) and danger-associated molecular patterns (DAMPs) or pathogen-associated molecular patterns (PAMPs), leading to caspase-1 activation [31,32]. Originally, pyroptosis was defined as caspase-1-induced monocyte death. It is now understood as a broader, gasdermin-mediated inflammatory cell death involving the activation of caspase-4/5/11 and affecting various cell types beyond monocytes [31]. Current research suggests that caspase-1 and caspase-4/5/11 are only exclusive to this process (Figure 1).

Conversely, caspase-2, caspase-7, and caspase-10 are only associated with apoptosis [33]. Preclinical and clinical evidence indicate that metabolic disturbances trigger nucleotide-binding oligomerization domain, leucine-rich repeat, and pyrin domain-containing protein 3 (NLRP3) inflammasome activation, leading to pyroptosis of pancreatic β-cells [34]. Indeed, sustained hyperglycemia promotes excessive ROS production, which acts as a key signal activating the NLRP3 inflammasome/pyroptosis pathway, exacerbating the progression of diabetic nephropathy and neuropathy [35]. In line with this notion, a study by Zhan et al. showed that the expression levels of NLRP3, caspase-1, and IL-1β were significantly increased in STZ-treated diabetic rats [36]. Similarly, Li et al. found that NLRP3 inflammasome recognizes risk signals, activating GSDMD and caspase-1, which drive pyroptosis and trigger the release of pro-inflammatory cytokines such as IL-1β via inhibition of AMPK/SIRT1 and activation of the NF-κB axis (Figure 1). These results were confirmed by the TUNEL experiment that pyroptosis promotes the progression of DN [37]. Moreover, Che et al. reported that high glucose exacerbates neuronal pyroptosis in a mouse model of T2DM [38]. Furthermore, in the study performed by Xu et al., high fat promoted the activation of the TXNIP/NLRP3 inflammasome in a diabetic mouse model [39]. Nutritional strategies aim to block NLRP3 inflammasome-mediated pyroptosis as a novel approach to suppress chronic inflammation and improve the diagnosis and treatment of diabetic complications. Emerging preclinical studies showed that Tan IIA (20 mg/kg daily intraperitoneal (i.p.) injection significantly suppressed NLRP3-Caspase 1-mediated GSDMD cleavage, alleviating renal pyroptosis and inflammation in vitro and in vivo [40]. Renal damage induced by hyperglycemia is also prevented through the blockade of pyroptosis, achieved by inhibiting activation of the thioredoxin-interacting protein (TXNIP)/TRX1/NLRP3 inflammasome axis, caspase-1, and gasdermin (GSDM)-mediated plasma membrane perforation in podocytes and glomerular endothelial cells [41,42]. The antifibrotic effect of Tan IIA was also confirmed by Zeng et al. in a study on HK-2 cells with glucose-induced damage [43]. In addition, Wu and coworkers demonstrated that Tan IIA (20 μg/mL) alleviated NLRP3 inflammasome and pyroptosis by modulating oxidative stress, targeting TXNIP/TRX1 in high glucose-induced renal glomerular endothelial cell damage and in db/db mouse models [44]. The Tangshen formula (TSF) can repress pyroptosis by regulating the TXNIP-NLRP3-GSDMD signaling pathway in HK-2 cells [45]. Moreover, the Yi Shen Pai Du Formula (YSPDF) can upregulate the Nrf2/HO-1 signaling pathway and downregulate ROS and the expression of NLRP3, ASC, and caspase-1 in DN via inhibition of the TGF-β1/Smad pathway [46]. Acteoside demonstrated renoprotective effects in DN by regulating the PI3K/AKT/NF-κB signaling pathway and alleviating pyroptosis [47]. In addition, UA exerted inhibitory effects on the secretion of IL-1β, IL-18, caspase-1, pyroptosis, and NLRP3 by suppressing SUMO1-mediated SUMOylation in primary mouse GMCs and SV40-MES-13 cells [48]. The main component of pomegranate polyphenols, in particular punicalagin (20mg/kg), significantly alleviated DN in mice, and the effect is associated with downregulating the expression of nicotinamide adenine dinucleotide phosphate (NADPH) oxidase 4 (NOX4), inhibiting the TXNIP/NLRP3 pathway-mediated pyroptosis. This suggests its potential as an adjuvant to conventional therapy for complications of diabetes [49]. The dose of 20 μg/mL of total flavones of *Abelmoschus manihot* (TFA) effectively inhibited the activation of the NLRP3 inflammasome related to pyroptosis (GSDMD-NT, IL-1β, and IL-18) via suppression of the PTEN/PI3K/Akt pathway in podocytes under high glucose conditions dose-dependently [50]. A recent study performed by Feng et al. has shown that tangzu granule alleviated pyroptosis and neuroinflammation induced by high glucose through inhibition of the P2X7R/NLRP3 signaling pathway in rats with diabetic peripheral neuropathy [51]. Loganin, an iridoid glycoside isolated from the fruit Cornus officinalis, prevented pyroptosis in RSC96 Schwann Cells under high glucose exposure by inhibiting ROS generation and suppressing NLRP3 inflammasome activation [52]. Cynarin inhibited the assembly of the NLRP3 inflammasome by Nrf2-dependent expression to mitigate microglial pyroptosis and neuroinflammation [53]. A randomized, double-blind, placebo-controlled clinical study involving sixty patients with T2DM receiving 600 mg/d of butyrate in synergy with 10 g/d of inulin showed stimulated expression levels of miR-146a-5p and miR-9-5p and a stress antioxidant response with SOD and CAT enzymes via inhibiting pyroptosis by targeting TLR2 and NF-κB1 after 45 consecutive days [54]. Interestingly, the Jiedu Tongluo Tiaogan Formula (JTTF), composed of 441 compounds, predominantly alkaloids, flavonoids, phenols, and terpenoids, revealed multi-target effects on T2DM-associated pyroptosis, particularly via the NLRP3/caspase-1/GSDMD pathway. In diabetic mice, JTTF dose-dependently reduced fasting blood glucose, IR, and dyslipidemia, while restoring pancreatic β-cell morphology. Specifically, JTTF suppressed NLRP3 inflammasome activation, downregulated caspase-1 and GSDMD expression, and attenuated IL-1β/IL-18 release (Figure 1) [55]. Overall, the data show promising personalized nutritional strategies in inhibiting NLRP3 inflammasome assembly and suppressing downstream pyroptosis in diabetic nephropathy and neuropathy. Currently, there are no studies on MNP-induced pyroptosis in diabetes; we hypothesize that nutritional medicine may block ROS formation and NLRP3 inflammasome activation, which in turn lead to IR and the onset of diabetes and its major complications.

## 5. Personalized Nutrition Targeting the *Nfe2l2* Gene and Redox Resilience Signaling in Diabetes and Its Complications

**Figure 2:**
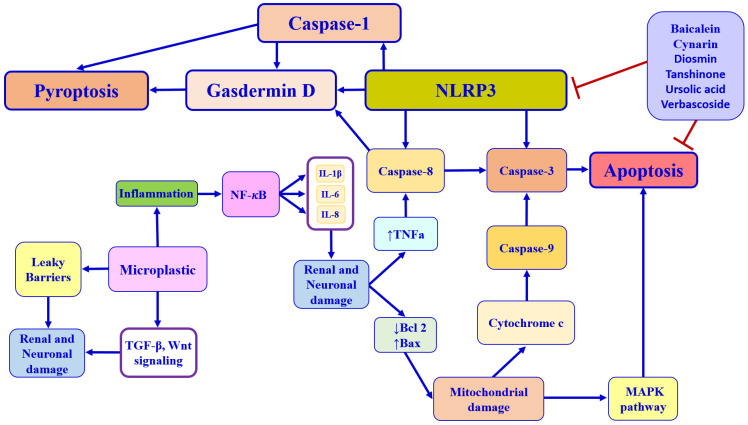
Molecular pathways involved in processes of apoptosis and pyroptosis mechanisms in cells. NLRP3—NLR family pyrin domain containing 3; NF-κB—nuclear factor kappa-light-chain enhancer of activated B cells; IL-1β—Interleukin-1 beta; IL-6—interleukin-6; IL-8—interleukin-8; TNF-α—tumor necrosis factor alpha; Bcl 2—B-cell lymphoma 2; Bax—Bcl-2-associated X protein; TGF-β—transforming growth factor-beta; Wnt—wing-less-related integration site; MAPK—mitogen-activated protein kinase. ↑ upregulation ↓ downregulation.

The unique physiological and genetic characteristics of individuals influence their reactions to different food nutrients. The notion of nutrient–gene interactions and the underlying molecular mechanisms that may explain the genetic basis of interindividual differences in response to specific nutritional compounds form the foundation of personalized nutrition. The emerging field of nutrigenetics has made significant progress in mapping how genetic variants (SNPs) affect nutrient metabolism and dietary responsiveness, enabling personalized, genome-guided, and precision nutrition strategies to optimize health and prevent environmental pollution challenges [56]. Functional nutrients can play a significant role in preventing and managing metabolic disorders [18]. By understanding how selective food nutrients/polyphenols, including flavonoids, flavanols, diterpenoids, triterpenoids, and dicaffeoylquinic acid derivatives modulate diabetes and its complications, such as diabetic nephropathy and diabetic neuropathy, personalized dietary interventions—knowledge of how the *Nfe2l2* gene and nutrients interact to optimize glycemic control and prevent disease onset—remain an underexplored field. According to recent scientific evidence, the consumption of these food nutrients can positively influence the human body by targeting the *Nfe2l2* gene and stress resilience proteins and enzymes [57,58,59,60,61]. Indeed, individuals with Nrf2 rs6721961 polymorphism have shown lower total anti-oxidative capacity associated with reduced β-cell function index, homeostasis model assessment, and increased risk of oxidative stress and diabetes. This genetic polymorphism may also contribute to impaired insulin secretory capacity and increased IR [62]. Intriguingly, food nutrients and/or nutraceutical supplements have been shown to prevent diabetes and its progression by targeting multiple pathways, specifically by blocking 5α-reductase, reducing IL-6 secretion, and inhibiting the lipid peroxidation process [57]. In this new light, recent evidence reported that natural exosome-like nanoparticles from mung bean sprout juice attenuate oxidative stress levels in the liver tissue of diabetic murine models. This is achieved by stimulating the PI3K/Akt pathway, which leads to the upregulation of GLUT4 and downregulation of GSK-3β, as well as by inducing the activation of the Nrf2 pathway and stress resilience enzymes, particularly HO-1 and SOD, in a dose-dependent and time-dependent manner [58]. Moreover, notoginsenoside (NG)-R1, a bioactive metabolite from *Panax ginseng*, significantly improves palmitic acid-induced IR by upregulating the Nrf2 pathway. This activation lowers oxidative stress, as indicated by a reduction of malondialdehyde (MDA) and 4-hydroxynonenal (4-HNE) in human umbilical vein endothelial cells [59]. Similarly, epiberberine attenuates oxidative stress and IR by enhancing stress resilience proteins and enzymes like SOD, GPX-Px, sNQO1, and HO-1 in both liver cells and the liver tissue of diabetic mice [60]. In addition, pomegranate peel polyphenols showed great potential and application prospects as functional foods or preventive drugs to improve pancreatic β-cell dysfunction. In particular, pomegranate peel polyphenols activate the PI3K/Akt pathway and promote the translocation of Nrf2 from the cytoplasm to the nucleus by upregulating FoxO1 expression, resulting in enhanced insulin signaling in vitro and in vivo [63]. An interesting randomized, double-blind, placebo-controlled study found that trans-resveratrol, in synergy with hesperetin, reversed IR in overweight and obese subjects by increasing the expression of glyoxalase 1 (Glo1). This increased Glo1 expression combats the accumulation of methylglyoxal and, via the induction of the Nrf2 pathway, decreases the expression of thioredoxin-interacting protein (TXNIP). This, in turn, helps reverse IR by counteracting the effects of dicarbonyl stress and improving glucose metabolism [64]. These redox-sensitive mechanisms provide a protective effect, and studies show these benefits are reversed if the *Nfe2l2* gene is silenced, highlighting its crucial role. Lastly, another double-blind randomized placebo-controlled clinical trial carried out on individuals with T2DM showed that supplementation with a dose of 2700 mg/day of omega-3 polyunsaturated fatty acids (PUFAs) enhanced Nrf2 gene expression and improved antioxidant capacity compared to placebo after a 10-week follow-up [65]. Thus, nutrients are effective in ameliorating oxidative stress and preventing T2DM complications. Collectively, these findings support the concept of nutrigenetics-based personalized nutrition targeting the *Nfe2l2***/***Nrf2* gene and redox resilience proteins and enzymes, which may offer a promising approach to prevent or manage diabetes by reducing oxidative stress and improving insulin sensitivity. Food nutrients can activate the Nrf2 pathway to mitigate hyperglycemic damage. This strategy is crucial because Nrf2 activity is often downregulated in diabetes. The nutrient selection, carried out according to the previously defined criteria, includes baicalein, cynarin, diosmin, tanshinone, ursolic acid, and verbascoside. Figure 2 illustrates the molecular processes involved in the mechanisms of apoptosis and pyroptosis in cells.

### 5.1. Baicalein

Baicalein (5,6,7-trihydroxyflavone 7-O-beta-D-glucuronide) (BA), a compound extracted from *Scutellaria baicalensis* roots, is an isoflavone with numerous pharmacological activities, including antioxidant, anti-viral, anti-diabetic, and anti-cancer effects (Figure 2) [66].

#### 5.1.1. Diabetes

Recent evidence demonstrated that BA effectively alleviates cytotoxicity and may represent a novel nutritional approach for managing metabolic diseases by targeting the *Nfe2l2* gene, offering a safer alternative to conventional therapies [67,68,69]. Numerous scientific studies have demonstrated the antidiabetic potential of BA, which acts through the synergistic modulation of the key mechanisms involved in glucose homeostasis. Indeed, BA treatment (10 mg/kg bw/day and 20 mg/kg bw/day) preserved renal function through anti-hyperglycemic, antioxidant, and anti-inflammatory effects by suppressing the activation of NF-κB, decreasing the expression of iNOS and TGF-β1 in the renal tissues of diabetic rats [68]. In a recent review, Qiu et al. compiled the results of a series of studies aimed at demonstrating the hypoglycemic efficacy of this compound and the main mechanisms involved, including increased glucose uptake, inhibition of gluconeogenesis, and improvement of IR [70]. First and foremost, these studies revealed that baicalin is capable of increasing cellular glucose uptake both in vitro and in vivo, as shown by the enhanced glucose utilization in insulin-resistant HepG2 cells, as well as increased glycolysis and hepatic glycogen content in animal models. These effects have been associated with the activation of the AMPK pathway, which promotes the translocation of the GLUT4 transporter from the cytoplasm to the plasma membrane via the phosphorylation of AS160, thereby improving glucose uptake in peripheral tissues, including adipocytes and myoblasts. Through the activation of AMPK, as well as the downregulation of both p38MAPK and STAT3, which in turn are regulated by the downregulation of Sirt1, BA suppresses the expression of genes involved in endogenous glucose secretion, thus inhibiting hepatic gluconeogenesis. Moreover, BA can enhance insulin secretion by stimulating AKT and inducing the expression of GLUT2 and glucokinase in pancreatic β-cells, thereby improving glucose uptake and metabolism. These effects were further confirmed in a study conducted by Nagarajan et al. on male Wistar albino rats with T2DM induced by streptozotocin [71]. In particular, following oral administration of 50 mg/kg of BA, a significant increase in insulin secretion, an improvement in total hemoglobin levels, and a marked reduction in blood glucose and glycated hemoglobin were observed compared to the control group. Additionally, BA contributes to improved insulin sensitivity and reduced IR, exerting insulin-sensitizing effects in peripheral tissues [70]. In this way, insulin responsiveness is enhanced in skeletal muscle and adipose tissue, again through AMPK activation. Finally, the compound plays a key antioxidant and anti-inflammatory role. By reducing oxidative stress and apoptosis, it protects pancreatic β-cells and preserves their function. It is noteworthy that the reduction of mitochondrial oxidative stress through activation of the KEAP1-Nrf2 axis makes BA potentially useful in preventing and counteracting both the microvascular and macrovascular complications of diabetes. This flavonoid not only modulates glucose metabolism, insulin sensitivity, and secretion, but also influences lipid metabolism and the inflammatory response, contributing to a more comprehensive management of T2DM [72]. Specifically, in mouse models fed a high-fat diet (HFD), intraperitoneal administration of 80 mg/kg/day of BA led to a significant reduction in body weight, serum free fatty acids, cholesterol levels, and tumor necrosis factor-α (TNF-α). These effects indicate that a dose of 5 and 10 mM of BA promoted anti-obesogenic and anti-inflammatory potential mediated via the activation of AMPK and acetyl-CoA carboxylase, resulting in reduced hepatic lipid accumulation, as confirmed in HepG2 cells treated for 24 h [73]. These effects indicate the anti-obesogenic and anti-inflammatory efficacy of BA, also mediated by the activation of AMPK and acetyl-CoA carboxylase, resulting in reduced hepatic lipid accumulation, as confirmed in HepG2 cells after 24 h of treatment. Furthermore, supplementation with 400 mg/kg/day of the aglycone form of BA reduced systemic inflammation, hyperglycemia, IR, and hyperlipidemia in HFD mice through the suppression of lipogenic genes (FAS and SREBP-1c) and the activation of PPAR-α, which is involved in fatty acid oxidation. In summary, due to its well-documented ability to coordinately modulate the mechanisms underlying various metabolic alterations by influencing insulin sensitization and reducing hyperglycemia-induced damage, BA could represent a promising multifunctional compound for the prevention and management of diabetes mellitus.

#### 5.1.2. Diabetic Nephropathy

BA appears to exert nephroprotective effects primarily by targeting antioxidant and anti-inflammatory pathways. Notably, it protected renal function by inhibiting the expression of AMPKα and inflammatory mediators, hs-CRP, and FcγR, in DN [74]. The combination of the three groups of herb components produced anti-DN effects through downregulation of inflammation mediated by NF-κB in rats [75]. An integrated study showed that the Qingxin Lianzi Yin Decoction, rich in active compounds like BA, liquiritigenin, succinic acid, formononetin, and wogonin, can potentially treat DN, probably related to glucose and lipid metabolism, oxidative stress, and inflammation [76]. In vivo and in vitro studies, respectively, on *db*/*db* mice and HK-2 cells, have shown that moderate doses of BA (25 mg, 50 mg, and 100 mg) have beneficial effects against DN by inhibiting the SphK1/S1P/NF-κB signaling pathway, which is involved in the progression of kidney damage in a concentration-dependent manner [77]. By blocking this pathway in *db***/***db* mice, BA inhibited inflammation, oxidative stress, blood glucose, and blood lipid levels, while improving renal function and reducing histological damage. Through the same mechanism, the flavonoid also reduced inflammation, oxidative stress, and apoptosis in HK-2 renal cells exposed to high glucose concentrations. In another study on *db***/***db* mice, Ma et al. demonstrated that BA improved general diabetic conditions by reducing proteinuria and renal cell apoptosis and protecting kidney tissue from histopathological changes [78]. Overall, BA provided nephroprotection by increasing renal levels of antioxidant enzymes (GSH-PX, SOD, and CAT) and concurrently reducing levels of MDA. It also promoted activation of the Nrf2 pathway, thereby stimulating the expression of the antioxidant enzymes HO-1 and NQO-1, suggesting an enhancement of the endogenous antioxidant response. At the same time, the compound exerted marked anti-inflammatory activity by significantly reducing renal infiltration of pro-inflammatory immune cells, including T lymphocytes, helper T cells, neutrophils, and macrophages, as well as the mRNA levels of pro-inflammatory mediators, including interleukin-1β (IL-1β), interleukin-6 (IL-6), monocyte chemoattractant protein-1 (MCP-1), and TNF-α. Renal transcriptomic analysis revealed that BA’s anti-inflammatory activity is also attributable to the inhibition of pro-inflammatory MAPK signaling pathways such as Erk1/2, JNK, and p38. Furthermore, in the same murine model, BA slowed podocyte injury by inhibiting the PI3K/Akt/mTOR signaling pathway, in addition to reducing creatinine, blood urea nitrogen (BUN), albuminuria, and improving renal histology in a dose-dependent manner [79]. Zhang et al. demonstrated that low doses of BA attenuate renal fibrosis, both in mice with streptozotocin-induced DN and in high glucose-treated HK-2 cells [80]. This effect results from inhibition of the TLR4/NF-κB pathway and the upregulation of miR-124, leading to a reduction in fibrotic markers in both experimental models. Conversely, high doses of BA (400, 800, and 1600 mg/kg/day) induce significant kidney injury and fibrosis through activating TGF-β/Smad signaling pathway [81]. Lastly, a clinical trial conducted on 95 patients with DN randomized to receive conventional treatment with or without BA for six months observed that this compound did not significantly alter fasting blood glucose or HbA1c levels. However, it significantly reduced urinary markers (microalbuminuria, urinary β2-microglobulin, urinary albumin excretion rate) and inflammatory biomarkers (NF-κB, vascular endothelial growth factor or VEGF), while increasing antioxidant enzyme activities (SOD and GSH-PX) compared to controls [82]. The findings from this and other cited studies demonstrate that BA can improve renal function in patients with DN, delaying its progression primarily through its anti-inflammatory and antioxidant actions. Taken together, the above findings demonstrate that BA promotes dose-dependent effects on renal health, acting as a renoprotective agent at low/mild doses by activating Nrf2 signaling, scavenging ROS, and reducing inflammatory cytokines. Conversely, high doses of BA can promote renal injury and fibrosis by activating the pro-inflammatory cascade and increasing oxidative stress markers.

#### 5.1.3. Diabetic Neuropathy

Growing evidence reported that BA appears to be a promising neuroprotective agent, capable of effectively modulating key processes involved in the pathogenesis of various forms of diabetic neuropathy. In particular, in streptozotocin-induced diabetic neuropathic pain models in rats, a single dose of BA (40 µg/kg, i.p.), as well as cumulative administrations, reduced the development of neuropathic pain in a dose-dependent mode [83]. This effect was mainly attributed to the suppression of the transient receptor potential vanilloid 1 (TRPV1) expression in the dorsal root ganglia. Furthermore, in a murine model of diabetic cardiac autonomic neuropathy, the flavonoid reduced the activity of the P2Y12 receptor in the stellate ganglia, normalized heart rate variability, and decreased satellite glial cell activation via p38 MAPK signaling [84]. This contributed to a reduction in neuronal inflammation and an overall improvement in neuropathic manifestations caused by chronic hyperglycemia typical of diabetes mellitus. In another study using a murine model of streptozotocin-induced diabetic peripheral neuropathy, Stavniichuk et al. showed that a dose of 30 mg/kg/i.p. of BA for four weeks improved motor and sensory nerve conduction velocity, as well as thermal and tactile sensitivity [67]. The observed effect was linked to reduced phosphorylation of p38 MAPK, a mediator of neuroinflammation and neuronal damage, decreased nitrosative stress, and inhibition of 12/15-lipoxygenase activity, which led to lower levels of its pro-inflammatory metabolite 12(S)-HETE. Therefore, although the molecule did not influence sorbitol accumulation or glucose metabolism intermediates in this study, it still exhibited a considerable neuroprotective effect by modulating inflammatory and oxidative pathways.

### 5.2. Cynarin

Cynarin is a key polyphenolic compound present in artichoke (*Cynara scolymus* L.). Specifically, it is a dicaffeoylquinic acid derivative found in the leaves and heads of the plant and is known for its antioxidant, anti-diabetic, and hepatoprotective potential (Figure 2) [85].

#### 5.2.1. Diabetes

Preclinical evidence showed the antioxidant capacity and the inhibitory effects of cynarin (1.0 mg/mL) found in *Helianthus annuus* against the formation of AGEs, which are rated as stronger than aminoguanidine (1 mM), a well-known synthetic antiglycative agent [85]. Synergistically, a combination of polyphenols contained in *Sorbus aucuparia* L., including caffeic, ferulic acid, chlorogenic acid, and cynarin, significantly inhibited the formation of AGEs, protected plasma proteins and lipids against nitration and oxidation, increased the non-enzymatic antioxidant capacity of plasma, and neutralized multiple oxidants generated in vivo. These stress resilience effects support the use of natural supplements as adjuvants to traditional therapies in T2DM and its complications [86]. In addition, the dose of 100 mg/kg/day *Cynara scolymus* leaf and *C. scolymus* flower for 28 days significantly improved the impaired hepatic function and markers like glycogen content, glycogen phosphorylase, glucose-6-phosphatase activities, serum fructosamine levels, lipid profile, aspartate transaminase activities, and alanine transaminase activities by suppressing inflammation and oxidative stress and enhancing GPx and SOD activities in diabetic rats. Of importance, the study revealed that the improvements may be attributed to their constituents, including phenolic acids, cynarine isomers, flavones, cynarasaponin isomers, hydroxyoctadecadienoic acid, and linolenic acid [87]. Moreover, artichoke water extract at a concentration of 0.25 and 2.5 mg/mL improved glucose metabolism by upregulating IRS1/PI3K/AKT/FoxO1 and GSK-3β signaling associated with the inhibition of endoplasmic reticulum (ER) stress and IR in HepG2 cells induced by palmitate dose-dependently [88].

#### 5.2.2. Diabetic Nephropathy

Cynarin is considered potentially useful in the treatment of DN due to its antioxidant, hypoglycemic, and hypolipidemic benefits, as previously discussed [89]. Ben Salem et al. tested the efficacy of an ethanolic extract from artichoke leaves, rich in cynarin, on Wistar rats with alloxan-induced diabetes (150 mg/kg). A 28-day treatment with doses of 200–400 mg/kg caused a significant reduction in blood glucose, total cholesterol, triglycerides, and LDL-C in the treated diabetic rats. At the same time, increased CAT, SOD, and GSH activity indicates an enhanced antioxidant defense system, which effectively mitigates oxidative stress, leading to a marked histological improvement at the renal, hepatic, and pancreatic levels [90]. The same dosage, administered for 60 days to Wistar rats fed a high-fat diet, was shown to reduce renal organomegaly and the levels of kidney markers such as creatinine and urea. It also helped to attenuate oxidative stress, thereby preserving renal histological integrity [91]. Therefore, although human clinical trials confirming the direct effect of cynarin on DN are currently lacking, the studies reviewed so far suggest that it could contribute to slowing the progression of the condition, whose main promoters are hyperglycemia, dyslipidemia, and oxidative stress.

#### 5.2.3. Diabetic Neuropathy

Experimental evidence on the effectiveness of cynarin in the treatment of diabetic neuropathy is limited. However, it is plausible to suggest that cynarin may help mitigate its symptoms due to the well-documented hypoglycemic, hypolipidemic, antioxidant, and anti-inflammatory properties attributed to *Cynara scolymus* L. By acting on the various factors underlying diabetes, the compound may prove useful in preventing diabetic complications, including neuropathy. The reduction of inflammation and oxidative stress, which are key contributors to nerve cell damage, is associated with improved nerve function and a reduction in neuropathic pain related to the disease. Haghighat et al. evaluated the antinociceptive effect of a hydroalcoholic extract from artichoke leaves in male rats with chronic constriction injury (CCI) of the sciatic nerve. The extract was administered via gastric gavage at doses of 200, 400, and 800 mg/kg for 21 days [92]. Behavioral tests conducted after 1, 4, 7, 14, and 21 days showed that the highest dose resulted in a significant reduction in thermal hyperalgesia, mechanical allodynia, and tactile allodynia, with effects observed from day 7 to day 21 of treatment. Biochemical analysis revealed that the extract significantly reduced lipid peroxidation and restored antioxidant enzyme activity (SOD and GPx) in sciatic nerve tissue, thereby alleviating neuropathic pain induced by elevated oxidative stress. Sciatic nerve injuries are frequently associated with diabetic neuropathy, as demonstrated by Schwarz et al. [93]. In this study, using magnetic resonance neurography (MRN), as well as morphological and proteomic analyses of sciatic nerves from patients with T2DM, extensive axonal degeneration, demyelination, and blood–nerve barrier disruption were observed. By targeting the protective and pathological mechanisms involved in diabetes, *Cynara scolymus* L., through its active components, including cynarin, could be considered a promising preventive strategy in the treatment of diabetic neuropathic pain. Collectively, these molecular processes, correlated to diabetes, are reported in Table 1.

### 5.3. Diosmin

Diosmin (3′,5,7-trihydroxy-4′-methoxyflavone-7-ramnoglucoside) is a flavonoid derived from citrus fruits like oranges and lemons. It regulates several biomarkers and pathways, including blood glucose, lipid profiles, and renal function, mainly by reducing oxidative stress and inflammation and enhancing cellular stress resilience response (Figure 2) [94].

#### 5.3.1. Diabetes

Preclinical studies demonstrated the potential inhibitory effects of diosmin against the enzymes aldose reductase, α-glucosidase, and α-amylase involved in diabetes and its complications, compared to the standard drugs in vitro [95]. A synergistic combination of luteolin and diosmin in selenium nanoparticles, 10 mg/kg for 6 weeks, has shown prominent anti-diabetic potential by enhancing the insulin levels and antioxidant enzymes in the kidneys and livers of diabetic mice [96]. Interestingly, a mild dose of diosmin (50 and 100 mg/kg, p.o) ameliorated glucose intolerance and the serum markers of liver function (alanine aminotransferase, aspartate transaminase, and alkaline phosphatase), as well as decreased fasting blood sugar against sodium arsenite-induced toxicity in mice [97].

#### 5.3.2. Diabetic Nephropathy

Research on DN has identified diosmin as a molecule useful in effectively counteracting several of the pathogenic mechanisms involved, where hyperglycemia is the triggering factor, initiating a plethora of harmful cellular and molecular events at the renal level. In this regard, Deng et al., in a study aimed at evaluating the efficacy of diosmin on HK-2 with hyperglycemia-induced damage, first observed how the high glucose concentration caused a significant reduction in cell viability and an increase in apoptosis, as evidenced by the increased expression of caspase-3 [98]. These effects were accompanied by a marked condition of endoplasmic reticulum (ER) stress, with elevated levels of GRP78 (a protein marker of ER stress) and upregulation of the CHOP protein, which is involved in the regulation of apoptotic genes and ROS production. However, there was an increase in NOX2 and MDA, along with a simultaneous reduction in the expression of the anti-apoptotic gene Bcl-2 and the activity of the antioxidant enzymes SOD, CAT, and MNSOD. Furthermore, hyperglycemia activated a strong inflammatory response through the stimulation of the NLRP3 inflammasome, with production of the pro-inflammatory cytokines, altered autophagic mechanisms, as indicated by increased levels of BECLIN1 and the LC3I/LC3II ratio, signs of cellular stress, and finally, reduced the expression of the nephroprotective gene PTEN. As a consequence, the PI3K/AKT pathway was activated, promoting tubular interstitial renal fibrosis through the inhibition of autophagy. However, co-incubation of HK-2 cells damaged by hyperglycemia with diosmin effectively counteracted all the aforementioned pathological mechanisms. The treatment restored the viability of renal cells by inhibiting apoptosis and caspase-3 expression, as well as attenuating oxidative stress and ER stress. Accordingly, reductions in MDA, GRP78, and CHOP levels were recorded, together with an increase in the antioxidant activity of SOD, CAT, and MNSOD and the expression of Bcl-2. The compound also exhibited pronounced anti-inflammatory and autophagy-regulating activity, counteracting the activation of the NLRP3 complex, thus inhibiting the expression of pro-inflammatory cytokines and normalizing the levels of BECLIN1 and LC3. Diosmin has been shown to counteract DN, contributing to improve the phenotype of renal podocytes, also through the inhibition of the PI3K/AKT pathway following PTEN overexpression [98]. The antifibrotic effect of diosmin was also confirmed by other studies. In particular, Zhao et al. demonstrated how the compound counteracted renal fibrosis by reducing the levels of inflammatory cytokines through activation of Sirt3 and the consequent inhibition of nuclear translocation of NF-κB p65 [99]. Ahmed et al. confirmed that this flavonoid showed significant nephroprotective effects in DN, counteracting renal damage caused by hyperglycemia-induced inflammation and oxidative stress [100]. Notably, diosmin treatment (50–100 mg/kg) for 28 days in Wistar rats with alloxan-induced DN reduced levels of NF-κB and pro-inflammatory cytokines, improved the activity of antioxidant enzymes (SOD, CAT, and GSH), and reduced levels of MDA and nitric oxide (NO) content [100]. Overall, the data highlights that dosmin exerts protective effects for preserving renal structure and function.

#### 5.3.3. Diabetic Neuropathy

Thanks to its strong antioxidant and anti-inflammatory properties, diosmin represents a potentially valid option for diabetic neuropathy, as confirmed by several researchers. Jain et al. studied the effect of oral administration of diosmin at concentrations of 50 and 100 mg/kg body weight for 4 weeks in male Sprague–Dawley rats with T2DM induced by streptozotocin and fed a high-fat diet [101]. At the end of the treatment, reductions in glycemic and lipid values were observed alongside a concurrent increase in body weight. However, the crucial aspect highlighted in this study is the molecule’s ability to counteract oxidative stress, reducing levels of MDA and NO while simultaneously increasing those of SOD and GSH. Thanks to the enhancement of antioxidant defenses, the development of early diabetic neuropathy was limited, as confirmed by reductions in thermal hyperalgesia, cold allodynia, and ambulatory difficulties in diabetic rats [101]. Gölboyu et al. suggested that diosmin, used alone or together with hesperidin, could improve motor function and promote nerve regeneration in diabetic Wistar rats by acting on fibroblast growth factor 21 (FGF21) and galectin-3 (gal3), important markers of oxidative damage and inflammation [102]. In particular, after treatment, the treated group showed, compared to controls, an increase in plasma levels of FGF21 and a reduction in plasma levels of galectin-3 and MDA, accompanied by a reduction in perineural thickness, as confirmed by histopathological examinations. Finally, in another murine model (male Swiss mice) with CCI, intraperitoneal administration of 1 or 10 mg/kg diosmin for 7 days reduced CCI-induced mechanical and thermal hyperalgesia by activating the NO/cGMP/PKG/KATP channel signaling pathway and inhibiting both spinal cord cytokine mRNA expression markers (IL-1β and IL-33/St2) and glial cell activation (microglia—Iba-1, oligodendrocytes—Olig2) [103]. Finally, a randomized controlled study demonstrated that oral supplementation of diosmin in synergy with hesperidin (1g/day each other) significantly reduced blood glucose, triglycerides (TGs), and low-density lipoprotein (LDL) from baseline [104]. In light of the mechanisms examined, the compound may prove very valuable in managing diabetic neuropathic pain.

### 5.4. Tanshinone

Tanshinones are a class of lipophilic diterpenoids from *Salvia miltiorrhiza*. They are classified by their molecular structure into cryptotanshinone (CT), tanshinone I (Tan I), tanshinone IIA (Tan IIA), and dihydrotanshinone (DT). Tanshinones exhibit various pharmacological activities, among which antioxidant, antibacterial, antidiabetic, antineoplastic, and antiangiogenic properties stand out (Figure 2) [105,106].

#### 5.4.1. Diabetes

Recent evidence indicates that Tan I shows potential as a therapeutic agent for T2DM by acting as an anti-inflammatory and insulin-sensitizing agent. It reduces the levels of IL-6 and TNF-α and inhibits nuclear translocation of NF-κB and IR by decreasing Ser307 phosphorylation in insulin receptor substrate 1 (IRS-1) in rats [107]. Network pharmacology studies predicted that the bioactive flavonoids found in *Pueraria Lobata Radix* and *Salviae Miltiorrhizae Radix*, particularly Tan IIA, in synergy with puerarin, formononetin, and luteolin, alleviated T2DM via targeting several molecular mechanisms and pathways, such as AKT1, VEGFA, NOS3, PPARG, MMP9, and VCAM1 [108]. The dried roots of *Salvia miltiorrhiza* Bunge are considered a useful remedy for treating various disorders, including hyperglycemia. This effect is mainly attributed to the lipid fraction, represented by tanshinones, which are therefore suitable for counteracting the alterations underlying diabetes mellitus. In particular, Carai et al. showed that *Salvia miltiorrhiza* extract containing 21% total tanshinones and 3.7% Tan IIA inhibited the rise in blood glucose levels in fasting healthy Wistar rats treated with a starch or glucose load dose-dependently [109]. The hypoglycemic effect observed can be attributed to various mechanisms triggered by the plant’s bioactive compounds. Specifically, it is well established that Tan IIA, Tan I, CT, and 15,16-dihydrotanshinone (DHTH) enhance phosphorylation and downstream signaling of the insulin receptor, thus exerting insulin-sensitizing effects. This is likely responsible for the DHTH-induced increase in glucose uptake, which also appears to inhibit gluconeogenesis. Beyond acting on glucose metabolism, Tan IIA also affects other factors associated with or promoting T2DM, such as hyperlipidemia and overweight. In particular, the molecule has been shown, in murine models, to reduce adipose mass and body weight, as well as the plasma levels of total cholesterol, low-density lipoprotein cholesterol (LDL-C), and TGs. Similarly, an increase in high-density lipoprotein cholesterol (HDL-C) was recorded. In an in vivo study on obese mice fed a high-fat diet, oral administration of 200/400 mg/kg/day of CT improved IR and reduced obesity via the activation of the AMPK signaling pathway, a key regulator of cellular energy metabolism [110]. Specifically, the treatment led to reductions in body weight, food intake, blood glucose and lipid levels, and improved insulin sensitivity, as evaluated through an intraperitoneal glucose tolerance test (IPGTT) and an intraperitoneal insulin tolerance test (IPITT). CT also promoted brown adipose tissue activation and white adipose tissue browning by increasing thermogenesis through the induction of uncoupling protein 1 (UCP1) and other brown adipocyte-specific genes. In vitro, the compound inhibited adipogenesis of adipose-derived mesenchymal stem cells by activating the AMPK pathway and inducing differentiation toward thermogenic phenotypes. This effect was enhanced by AMPK agonists and nullified by inhibitors, confirming the central role of this pathway. Additionally, CT appears to improve insulin responsiveness by targeting the PI3K/AKT pathway, thereby increasing glucose uptake by cells and counteracting IR, which often characterizes T2DM [111]. Of note, Yuan et al. showed that Tan IIA exerted protective effects in a murine model of T2DM by improving IR, glucose, and lipid metabolism, and reducing body weight [112]. Tan IIA may counteract chronic inflammation and improve metabolic control in T2DM by upregulating the AMPK pathway and downregulating NF-κB, thus reducing the levels of IL-6, IL-8, TNF-α, and C-reactive protein (CRP), key inflammatory mediators involved in the pathogenesis of T2DM [112]. Moreover, in diabetic rats treated with Tan-I, a marked reduction in blood glucose levels and improvement in lipid profile were observed, including decreases in total cholesterol, TGs, non-esterified fatty acids, and LDL cholesterol levels. Taken together, tanshinones act on the various metabolic and hormonal alterations associated with diabetes mellitus, making them a valuable natural remedy also for the management of its related complications.

#### 5.4.2. Diabetic Nephropathy

Several studies have shown that some of the lipophilic active components of *Salvia miltiorrhiza* possess antioxidant, anti-inflammatory, and immunomodulatory properties in DN [113,114,115,116]. In particular, in murine models of T2DM, Tan IIA counteracted inflammatory processes and improved metabolic dysfunction by inhibiting NF-κB and activating AMPK [112]. Simultaneously, it reduced proteinuria and podocyte foot process effacement and inhibited extracellular matrix deposition and fibroblast activation, thereby preventing the onset of renal fibrosis. Treatment with 6 μg/mL of Tan IIA counteracted glucose-induced renal fibrosis by inhibiting extracellular matrix deposition and the epithelial–mesenchymal transition, as confirmed by reduced expression of transforming growth factor-β1 (TGF-β1), α-smooth muscle actin (α-SMA), and laminin (LN), along with increased expression of E-cadherin (E-cad) [114]. Furthermore, Tan IIA increased the expression of HO-1, a cytoprotective enzyme that counteracts oxidative stress, thereby slowing the progression of DN [117]. Li et al. demonstrated that Tan IIA prevented podocyte damage by promoting autophagy and reducing inflammation through inhibition of the PI3K/Akt/mTOR signaling pathway in both male db/db mice and murine podocyte cells (MPC5) under high glucose conditions [118]. The regulation of the PI3K/Akt/NF-κB signaling pathway by Tan-IIA also contributes to reducing the renal damage typical of DN [119]. Additionally, in MPC5 under hyperglycemic conditions, the compound suppressed apoptosis, the inflammatory response, and ferroptosis by targeting the ELAVL1-ACSL4 axis [120]. Moreover, in streptozotocin-induced diabetic murine models, a dose of 2 and 4 mg/kg/day Tan IIA alleviated renal dysfunction and morphological alterations by suppressing protein kinase RNA (PKR)-like ER kinase (PERK) pathway activation and collagen expression via targeting SOD activity [121]. Synergistically, salvianolic acid B and Tan IIA improved glucose and lipid disorders, liver and kidney damage, and blocked kidney inflammation in early DN rats by regulating the PI3K/AKT/NF-κB signaling pathway [119]. Tan IIA at doses of 5, 10, and 25 mg/L improved DN-induced renal fibrosis by upregulating miRNA-34a-5p and directly targeting Notch1 in vitro and in vivo [122]. Tan IIA attenuated TNF-α, IL-1β, IL-6, and IL-18 and uric acid-induced NLRP3 inflammasome formation by targeting the Nrf2 signaling pathway in vivo [123]. Finally, Tan IIA (10 or 30 mg/kg/day) enabled the clearance of the toxic intermediate metabolite NAPQI from the kidney via the upregulation of the Nrf2-MRP2/4 pathway in vitro [124]. Overall, Tan IIA may provide preventive and therapeutic effects in combination with traditional therapy against nephrotoxicity in DN.

#### 5.4.3. Diabetic Neuropathy

Emerging studies suggest that the efficacy of *Danshen* in the treatment of diabetic peripheral neuropathy is primarily due to its anti-inflammatory and anti-apoptotic properties, which act mainly through the modulation of AGE–RAGE and PI3K–AKT signaling pathways [125]. In particular, Tan IIA displays significant anti-inflammatory activity, making it a promising nutritional strategy for alleviating the pain associated with the diabetic condition. Feng et al. demonstrated that Tan-IIA alleviated neuropathic pain in diabetic rats by activating the Nrf2/ARE signaling pathway and inhibiting the NF-κB signaling pathway [126]. Moreover, Zhang et al. observed that a dose of 40 mg/kg/day of this lipophilic diterpene for 3 weeks reduced thermal hyperalgesia and mechanical allodynia in streptozotocin-induced diabetic rats by repressing inflammation [127]. Specifically, the treatment reduced the expression of IL-1β, IL-6, and TNF-α in the spinal ganglia and increased IL-10 levels, a key anti-inflammatory cytokine, thereby relieving pain associated with inflammation. Additionally, according to results by Rigele et al., Tan IIA markedly reduced thermal hyperalgesia and mechanical allodynia in the same rat model by normalizing the activity and expression of voltage-gated sodium channels in the dorsal root ganglia [128]. These channel levels are elevated in diabetic rats and contribute to neuropathic pain. Another mechanism involved in peripheral diabetic neuropathic pain (DPNP) is the disinhibition of spinal dorsal horn neuronal circuits, mediated by endoplasmic reticulum stress [129]. Tan-IIA modulates this process, improving both mechanical and thermal thresholds in diabetic rats with DPNP. Finally, Liu et al. reported a marked neuroprotective effect in streptozotocin-induced diabetic rats following 4 weeks of treatment with Tan IIA [130]. This therapy led to significant enhancements in peripheral nerve function, evidenced by increased motor nerve conduction velocity and restoration of nerve blood flow, along with the recovery of thermal and mechanical nociceptive sensitivity. This diterpene also significantly improved the redox balance in the sciatic nerves by increasing SOD and CAT activity, while reducing MDA levels. Furthermore, it activated Na^+^/K^+^-ATPase, a crucial enzyme for neuronal function, whose activity is compromised under chronic oxidative stress, such as in diabetes. Total serum antioxidant capacity was also enhanced. Overall, findings from various studies on Tan IIA reveal its potential to counteract diabetic neuropathy by improving nerve function through multiple mechanisms, particularly by targeting antioxidant and anti-inflammatory pathways. Collectively, these molecular processes correlated to diabetes are reported in Table 2.

### 5.5. Ursolic Acid

Ursolic acid (UA), a natural pentacyclic triterpenoid found in apples, basil, berries, and fruit peels, has shown promising pharmacological potential for various chronic disorders. Recent research indicates its potential effects are related to its antioxidant and anti-inflammatory, antidiabetic, nephroprotective, and neuroprotective properties, which help to prevent or attenuate inflammation and oxidative damage [131] associated with chronic disorders by targeting the Nrf2 pathway and cellular resilience genes and enzymes (Figure 2). This is supported by both in vitro and in vivo studies that show its potential beneficial health effects in several organs, including the pancreas, liver, heart, and brain [132].

#### 5.5.1. Diabetes

Growing evidence has shown that low-dose UA (25 mg/kg p.o.) in synergy with roseus ethanolic extract (25 mg/kg p.o.) effectively increased antioxidant defense enzymes (e.g., CAT, SOD, GPx, and GSH) and reduced blood glucose levels after 28 days against streptozotocin-induced diabetes in rats [133]. In particular that dietary intake of UA is well-tolerated up to a dose of 9.26 g/kg, and its consumption can be considered safe, as suggested by an oral LD50 value greater than 8000 mg/kg in mice [134]. Furthermore, UA plus loquat leaf extract reversed hyperglycemia-induced hepatic inflammation by upregulating the LKB1-AMPK1-FOXO3 axis [135]. Moreover, UA at doses of 2.5, 5, and 10 mg/kg administered orally to the hyperglycemic rats for 8 weeks decreased oxidative stress in pancreatic tissue by enhancing GSH-Px and SOD activities, suppressing the Traf-6, Mapk-8, and Traf-4 mRNA expression and activating the expression of Pdx-1, Ins-1, and Ngn-3 to regenerate pancreatic β cells to secrete insulin [136]. Molecular-docking studies showed that UA binds the site of α-amylase and α-glucosidase through the formation of UA-glucosidase complex and inactivates them to reduce the postprandial blood glucose level in C57BL/6J mice [137]. UA lactone (50 µg/mL) obtained from *Eucalyptus tereticornis* increased glucose uptake in insulin-resistant muscle cells and reduced triglyceride content in hepatocytes and adipocytes, targeting the AMPK pathway [138]. Interestingly, supplementation with UA (250 mg) associated with exercise training decreased oxidative stress markers such as MDA and enhanced the Sirt1–endothelial nitric oxide synthase (eNOS) axis in diabetic rats [139]. Recent data demonstrated that triterpene acid complex/Se-methylselenocysteine significantly improved palmitic acid (PA)-induced IR in HepG2 cells via upregulating the PI3K/AKT/GSK3β pathway [140]. Of note, UA introduced into CS-PVA nanofiber dressings significantly enhances diabetic wound healing by acting as an anti-inflammatory and antioxidant agent. In vivo studies showed that nanofiber dressings decreased the release of TNF-α and IL-6 levels, lowered ROS-induced oxidative stress, promoted angiogenesis (revascularization), and improved re-epithelization [141]. Interestingly, UA from *Artemisia montana* significantly suppressed protein tyrosine phosphatase 1B (PTP1B) and α-glucosidase activities, as well as AGEs, and increased GLUT-4 expression by upregulating the IRS-1/PI3K/AKT/GSK-3 signaling pathway in insulin-resistant C2C12 muscle cells [142]. In addition, UA, the main bioactive compound found in the hydroalcoholic extract of *P. australis* at the doses of 100 and 50 mg/kg, showed antidiabetic activity by sensitizing insulin through PPARγ/GLUT-4 overexpression [143]. Likewise, the dose of 100 mg/kg of UA and oleanolic acid isolated from *Salvia polystachya* exhibited potent antihyperglycemic effects by reducing blood glucose levels and the postprandial peak after sucrose, thus inhibiting α-glucosidases similar to glibenclamide in vivo, ex vivo, and in silico [144]. Finally, a recent study by Thabah and colleagues demonstrated that UA in synergy with other flavonoids, such as catechin, epicatechin, kaempferol, metformin, and quercetin present in *Potentilla fulgens*, ameliorated hyperglycaemia and insulin sensitivity and increased the expression of GLUT4, AKT2, AMPKα1, and AMPKα2 via the activation of the AKT2 and AMPK signaling pathways [145].

#### 5.5.2. Diabetic Nephropathy

A physiological dose of 25 mg/kg of UA alleviated the inflammatory state by targeting the TLR4-mediated inflammatory pathway in murine models of DN [146]. Recent research demonstrated that UA effectively reduced proteinuria, infiltration of immune cells, and histopathological damage in the renal tissue. Interestingly, a diet containing 0.3 g of UA promoted a high preventive potential against DN progression by blocking the overactivation of the autophagic P62-mediated NF-κB p65-MDM2-Notch1 signaling pathway both in vivo and in vitro [147]. Moreover, UA in synergy with metformin reduced the levels of blood glucose, HbA_1C_ (glycated hemoglobin), creatinine, uric acid, blood urea nitrogen, AST (aspartate aminotransferase), ALT (alanine aminotransferase), and ALP (alkaline phosphatase), and AGEs’ formation in the plasma and kidney [148]. Other studies observed that low doses of UA (35 mg/kg) significantly inhibited TNF-α, monocyte chemoattractant protein-1 (MCP-1), and IL-1β and activated redox resilience enzyme SOD in the kidney of streptozotocin (STZ)-induced diabetic rats [149]. Finally, the synergistic combination of insulin and UA ameliorated the pathological changes by upregulating p38 MAPK, SIRT3, DPP-4, and FGFR1 levels, thereby blocking TGF-β signaling pathway activation and inhibiting the epithelial–mesenchymal transition (EMT) and endothelial–mesenchymal transition (EndMT) processes in the renal tissue of T1DM rats [150].

#### 5.5.3. Diabetic Neuropathy

The potent anti-inflammatory and antioxidant properties of UA, along with its ability to counteract peripheral nerve damage, make it a promising nutritional candidate for the treatment of diabetic neuropathy. As demonstrated by Bhat et al. in a murine model of neuropathic pain induced by CCI, oral administration of UA (5–20 mg/kg) for 14 days significantly reduced mechanical and thermal hyperalgesia by attenuating the release of pro-inflammatory cytokines and decreasing the markers of oxidative stress, such as MDA and carbonylated proteins [151]. UA also demonstrated activity as a free-radical scavenger by removing NO and superoxide radicals, reducing lipid peroxidation, and restoring glutathione reserves. Studies have shown that UA attenuates the symptoms of diabetic peripheral neuropathy by activating PPARγ, which is involved in the regulation of inflammation and lipid metabolism, and by inhibiting TRPV1, which mediates neuroinflammation and pain [152]. Moreover, UA offers neuroprotection by acting as a PPARγ agonist, thereby modulating apoptosis-related pathways associated with endoplasmic reticulum stress, reducing nerve degeneration and peripheral neuropathic pain [153]. Finally, long-term administration of UA (14.4 mg/day) inhibited Aβ42 and P-tau levels. This protective mechanism induced by UA reversed cognitive decline, increased locomotion, and improved working memory by upregulating the CREB-BDNF signaling pathway in C57BL/6 J mice after 12 months [154].

### 5.6. Verbascoside

Verbascoside (VB) is a water-soluble phenylpropanoid glycoside (acteoside) isolated from *Verbascum sinuatum* [155]. Structurally, it is composed of a hydroxytyrosol residue linked to caffeic acid, esterified to a disaccharide (rhamnose linked to glucose) through glycosidic and ester bonds [156].

#### 5.6.1. Diabetes

VB is known to regulate diabetic conditions by exhibiting potent antioxidant, anti-inflammatory, and hypoglycemic properties [157]. Therefore, it is being studied for its potential synergistic effects with conventional therapeutic applications (Figure 2) [158]. Polyphenolic metabolites, especially phenylpropanoid derivatives and glucuronidated flavonols, i.e., V, FE, HT, Q3G, and L7DG, were the most promising polyphenols of all those studied for reversing the alterations induced by hyperglycemia in SGBS human adipocytes, as they could modify two of the three parameters measured (protein expression, polar metabolites, and/or lipids) [159]. The major bioactive compounds of *C. glandulosum leaf* extract, particularly VB, showed antihyperglycemic and antioxidant potential in blocking oxidative stress-induced hyperglycemia and managing metabolic syndrome [160]. Several studies suggest the potential beneficial effects of VB in the prevention and treatment of diabetes mellitus, acting through various mechanisms. Galli et al. evaluated the protective role of the glycoside on murine (βtc3) and human pancreatic β-cells under normal conditions and endoplasmic reticulum stress (ERS), following co-incubation for 5 days at different concentrations (0.8–16 µM) [161]. The treatment reduced oxidative stress by modulating the PERK pathway of the unfolded protein response and promoted mitochondrial dynamics. In this way, it dose-dependently preserved both types of β-cells from ERS-induced dysfunction, enhancing their viability, mitochondrial function, and intracellular insulin content. Moreover, it is known that VB administration can effectively prevent oxidative stress and inflammation-induced damage in the islets of Langerhans by preserving β-cell function through reduced ROS production and attenuation of the NF-κB signaling pathway [162]. In addition, male db/db mice treated for 45 days with different doses of Cistanche tubulosa (equivalent to 120.9, 72.6, or 24.2 mg of VB/kg) showed a significant reduction in both fasting and postprandial blood glucose levels, as well as in weight loss. Improvements in IR and dyslipidemia have also been observed [163]. Likewise, the hydroalcoholic extract of *Plantago australis*, rich in VB, demonstrated antidiabetic effects. Specifically, in non-insulin-dependent diabetic mice, administration of the extract led to a reduction in blood glucose levels through the inhibition of α-glucosidase, and a marked overexpression of PPARγ and GLUT4, resulting in increased insulin sensitivity [143].

#### 5.6.2. Diabetic Nephropathy

A recent study showed that high glucose can directly promote glucose uptake through sodium–glucose transporter 2 (SGLT2). Therefore, VB is a potential health-promoting agent by reducing the AMPK/NOX4/NF-κB signaling cascade in human proximal tubular renal (HK-2) cells [157]. Moreover, VB prevented high glucose-induced HK-2 cells and diabetes in db/db mice by inhibiting the NADPH/oxidase-TGF-β/Smad signaling pathway [164]. Moreover, acteoside protected podocytes from apoptosis, decreased the urine albumin of db/db mice, and delayed the course of diabetic kidney disease through inhibition of the AKT/GSK-3β signaling pathway [165]. The effectiveness of VB in the treatment of DN has been the subject of various studies. These investigations have highlighted the antioxidant and anti-inflammatory properties of the VB molecule. In particular, Ahmed et al. evaluated the effect of the phenylethanoid glycoside on HK-2 exposed to high glucose conditions. They assessed glucose uptake and, therefore, SGLT2 transporter activity using 6-NBDG, a fluorescent glucose derivative [157]. The treatment significantly reduced glucose uptake by inhibiting SGLT2 and attenuated inflammation and fibrosis through modulation of the AMPK/NOX4/NF-κB signaling pathway. Specifically, VB counteracted the increase in NOX4-derived ROS, reduced NF-κB phosphorylation, and activated the AMPK pathway, thereby suppressing the expression of IL-6 and TNF-α and fibrotic proteins such as fibronectin and collagen IV. Zhou et al. investigated the mechanisms underlying the protective effect of acteoside on renal interstitial fibrosis in rats with streptozotocin-induced diabetes combined with unilateral nephrectomy. Compared to controls, acteoside-treated rats showed a significant reduction in renal fibrosis by decreasing ROS levels and regulating both autophagic flux and lysosomal function, processes altered in renal cells under hyperglycemic conditions, through the regulatory influence exerted on transcription factor EB (TFEB) [166]. Furthermore, in streptozotocin-induced diabetic mouse models, it has been demonstrated that VB counteracts DN by lowering the expression of inflammatory/fibrotic signals, including chemokine MCP-1 and TGF-β1 [167]. In light of these findings, VB can be considered a functional food nutrient to slow the progression of DN through its synergistic antioxidant, anti-inflammatory, and autophagy-restoring activities.

#### 5.6.3. Diabetic Neuropathy

Although specific studies on the role of VB in diabetic neuropathy are lacking, experiments on murine models of neuropathic pain have demonstrated that the molecule exerts analgesic effects, mainly by attenuating nociception through anti-inflammatory and antioxidant mechanisms. These mechanisms, as previously discussed, are central to the development of various diabetic complications [168,169,170]. Of note, VB significantly improves the severity of MPTP-induced peripheral dopaminergic neuropathy by targeting the upregulation of AKT expression and the downregulation of the caspase 3 (CASP3) pathway in murine models [171]. Therefore, the neuroprotective action of this phenylethanoid glycoside could potentially help mitigate diabetic neuropathy. Collectively, these molecular processes correlated to diabetes are reported in Table 3.

## 6. Food Nutrients Prevent and/or Reverse MNP Damage and Toxicity in Chronic Disorders

Since biodegradation processes accelerate the dispersion of MNPs [172], microscopic fragments of these particles can penetrate the food chain, jeopardizing the reliability of traditional plastic materials used in food packaging. Recent experimental evidence supports that MPs (50–100 μm) induce excessive ROS generation and cytotoxicity in caco-2 cells [173]. The same study also revealed that a dose ranging from 0.1 to 2.0 mg/mL of phenolic acids, mainly e.g., hydroxybenzoic acids and hydroxycinnamic acids and flavonoids (e.g., flavanols and flavonols), had a dose-dependent antioxidant potential in counteracting MP-induced ROS damage and acute toxicity in vitro [173]. A recent study has shown that a dose of 25 mg/L of resveratrol butyrate esters administered via drinking water in young rats reversed MP exposure-induced hypertension by modulating both the classical and nonclassical renin–angiotensin system, as well as NO, which contribute to its protective effects. Furthermore, this study also observed that resveratrol butyrate esters altered gut microbiota composition, increasing butyric acid levels and elevating renal GPR41 expression [174]. Moreover, curcumin has been shown to mitigate MNP-induced toxicity through early induction of the Nrf2/ARE antioxidant pathway and inhibition of the NF-κB-mediated proinflammatory response [175]. In addition, another study reported that epigallocatechin-3-gallate (EGCG), the main polyphenolic constituent of green tea, promoted the increase in probiotic flora and effectively attenuated microplastics (MPs)-induced hepatic and colonic inflammation in mouse models [176]. A recent finding by Kavinda et al. suggested that anthocyanin-rich polyphenols isolated from *Hibiscus syriacus* L. extracts significantly reversed the osteogenesis impairment caused by polystyrene microplastics through autophagy activation. This effect was demonstrated by increased LC3B levels and a concomitant reduction in p62 expression in zebrafish models [177]. It is noteworthy that PS-NPs tend to accumulate in the intestinal lumen, causing tissue structural alterations and compromising barrier function. Furthermore, they interfere with gene expression in the small intestine, responsible for the deregulation of the immune response, and induce a state of intestinal dysbiosis. In line with these findings, quercetin administration (50 mg/kg) attenuated PS-NP-induced tissue damage at the intestinal level and immune dysfunction, normalizing gene expression and serum insulin levels, and ultimately promoting microplastic excretion in murine models [178]. Finally, another recent study suggested that glycyrrhizic acid and tannic acid at concentrations of 0.1 g/L and 1 g/L attenuated PE-MP-induced oxidative stress and toxicity by modulating the expression of the *gst-4* gene (encoding glutathione S-transferase-4), an enzyme involved in detoxification processes in *C. elegans* dose-dependently [179]. Currently, very few studies investigate the harmful effects of MNPs and the potential protective effects of food nutrients/polyphenols in counteracting them. There are no clinical studies directly evaluating the therapeutic potential of food nutrients to counteract MNP toxicity in humans. Overall, preclinical data suggest that MNPs induce damage in many cells, tissues, and organs, resulting in the risk of developing chronic diseases. Personalized nutritional interventions aimed at mitigating the adverse effects of human exposure could reduce the toxic effects of MNPs, which warrants further investigation and validation.

## 7. Role of Food Nutrients as Epigenetic Modulators Targeting *Nfe2l2* Gene: Nutriepigenomics Approach

**Figure 3:**
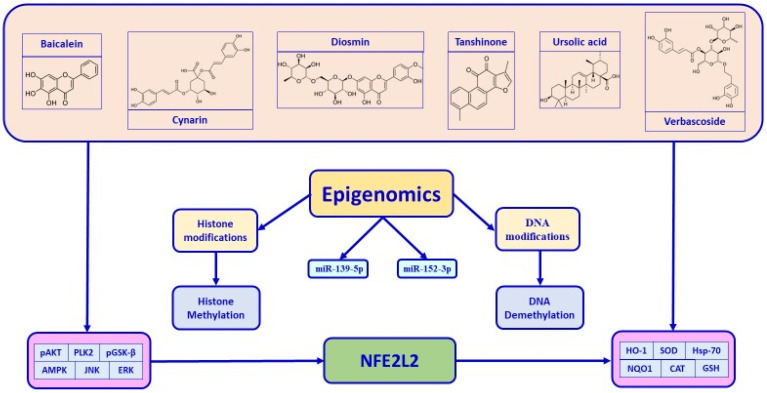
Schematic representation of the interaction between dietary bioactive compounds and epigenetic regulation. Activation of *Nfe2l2* gene and cellular stress resilience signaling cascade in epigenetic changes. pAKT—phosphorylated AKT; PLK2—polo-like kinase 2; pGSK-β—phosphorylated glycogen synthase kinase 3 beta; AMPK—5′ AMP-activated protein kinase; JNK—c-Jun *N*-terminal kinase; ERK—extracellular signal-regulated ki-nase; miR-139-5p—microRNA-139-5p; miR-152-3p—microRNA-152-3p; NFE2L2—nuclear factor erythroid-derived 2-like 2; HO-1—heme oxygenase-1; SOD—superoxide dismutase; Hsp-70—heat shock protein 70; NQO-1—NAD(P) H:quinone oxidoreduc-tase1; CAT—catalase; GSH—glutathione.

Nutriepigenomics is an emerging field of nutritional personalized science that studies how nutrients and dietary patterns influence gene expression through epigenetic modifications [180]. In the context of diabetes, this field is particularly relevant due to the significant impact of diet on disease management and the prevention of associated complications. The importance of nutriepigenetics in determining the minimum physiologically effective doses of bioactive food nutrients to inhibit DNA methyltransferases (DNMT) and histone deacetylase (HDAC) activity through the upregulation of the *Nfe2l2* gene represents an essential mechanism in disease prevention and management, which is still unexplored. The recent literature has shown that food nutrients and nutraceutical compounds can modify epigenetic patterns and, consequently, influence glucose metabolism by regulating the genes involved in insulin sensitivity and glucose production, chronic inflammation characteristic of diabetes (which can be modulated by anti-inflammatory nutrients), and oxidative stress through dietary antioxidant interventions that may reduce oxidative damage in metabolically active tissues [181]. Research conducted on male C57BL/6J mice indicates that epigenetic silencing of Nrf2 impairs kidney function and promotes hypertension. This process occurs through a reduction in resilience enzymes, which triggers increased oxidative stress and inflammation [182]. Dimethyl fumarate (DMF) has been shown to accelerate diabetic wound healing by activating the Nrf2/HO-1 signaling axis. This biochemical pathway counteracts the oxidative stress caused by hyperglycemia and downregulates the synthesis of pro-inflammatory mediators in macrophages exposed to high glucose concentrations with 25 mM [183]. In parallel, the ERK signaling pathway participates in the inhibition of Nrf2 function at the cardiomyocyte level, a phenomenon related to IR triggered by oxidative processes [184]. Of note, the impairment of the Nrf2/ARE system determines an increase in the oxidative load, while alterations in mitochondrial functionality end up triggering both IR and the endothelial abnormalities typical of diabetic pathology [185]. It is noteworthy that several studies have highlighted that, in Nrf2 knockout mouse models, insulin release from pancreatic islets is deficient, whereas, on the contrary, the increase in Nrf2 levels is able to enhance the secretory capacity of pancreatic β cells [186]. Finally, in mice with streptozotocin (STZ)-induced diabetes, sulforaphane administration successfully stimulated Nrf2 activity, counteracting the development of nephropathy and significantly normalizing metabolic markers such as hyperglycemia, polydipsia, polyuria, and weight loss related to type-2 diabetes [187]. Moreover, quercetin appears to affect many factors and cellular pathways involved in IR and the pathogenesis of T2DM and its complications, including TNFα, NF-KB, AMPK, AKT, and Nrf2 [188]. Also, the main polyphenols in agrimony, named agrimonolide and desmethylagrimonolide, lead to increased Nrf2/HO-1 expression and reduced p38 MAPK expression to attenuate the oxidative stress model in HepG2 cells [189]. Similar results indicated that low doses of 20 and 40 mg/kg of sinapic acid ameliorated the increased level of glucose, lipid, and cardiac function by upregulating Nrf2/HO-1 and downregulating NF-ΚB in STZ-induced diabetic rats [190]. Finally, the flavonoid vitexin protected endothelial cells from high glucose-induced damage via activation of the Wnt/β-catenin and Nrf2 signaling pathway (Figure 3) [191].

Diabetes mellitus is characterized by increased blood glucose levels. Glucose overload affects the maintenance of cellular homeostasis, particularly at the mitochondrial level, increasing free radicals, which subsequently alter the redox balance and lead to oxidative stress [192]. In diabetic subjects, oxidative stress can lead to the onset of complications, such as vascular alterations: micro-and macroangiopathies. Specific research has shown that the kinases regulated by the extracellular signal (ERK) act as a negative regulator of glucose absorption, mediating the resistance to insulin induced by oxidative stress [193]. Key regulators, such as KEAP1 and CUL3, are involved in the *Nfe2l2* gene/Nrf2 pathway. The dysregulation of these molecules, which govern cellular responses through redox-sensitive signaling networks, is implicated in diabetes, cancer, and neurodegeneration. The reduced activity of the Nrf2 pathway increases oxidative stress and mitochondrial dysfunctions, with consequent IR and endothelial dysfunction [194]. Studies on knockout mice for Nrf2 have detected a decrease in the production of insulin from the pancreas, while the overexpression of Nrf2 has increased the pancreatic production of insulin [184]. It is known that gene transcription is influenced by epigenetic modifications, which act through alterations in chromatin structure. This process ensures a modulation of cellular activity in response to external stimuli. Modifications, which include chromatin remodeling, DNA methylation, and the regulation of noncoding RNAs, are intrinsically reversible. Different classes of epigenetic modifications can regulate gene expression, playing a key role in essential biological processes such as cell development and differentiation, and adaptive responses to stress [185]. The dynamic nature of this type of alteration allows for modulation of the activity and stability of the *Nfe2l2* gene. Concurrently, methylation of CpG islands located in the *Nfe2l2* gene promoter acts as a repressive process. This mechanism suppresses Nrf2 transcriptional activity, resulting in a decrease in the transcription factor’s expression levels and, consequently, compromising the cell’s antioxidant capacity [186]. Recent research has highlighted how bioactive compounds of plant origin are able to modulate the expression of the Nrf2 factor by regulating these epigenetic modification mechanisms. Specifically, some bioactive compounds from food sources have been identified as inhibitors of DNMTs, enzymes that are part of the epigenetic system. This interaction results in the hypermethylation of CpG islands located in the *Nfe2l2* gene promoter. The biological effect is an upregulation of Nrf2 protein levels, whose increased expression confers protective benefits in pathological conditions associated with oxidative stress [195]. Demethylases, a class of enzymes known as TET proteins, are capable of demethylating DNA, in which cytosines undergo a process that involves the removal of methyl functional groups. When demethylation occurs specifically in the promoter region of the *Nfe2l2* gene, the block that prevents gene activation is removed. Decreased methylation levels in this region facilitate access to transcription factors and promote the binding of regulatory proteins to the promoter. This sequence of events increases the transcription and expression of the Nrf2 protein. Increased Nrf2 levels allow cells to strengthen their antioxidant defenses against oxidative stress, potentially improving the progression and prognosis of some oxidative stress-related diseases [195]. Noncoding RNAs (ncRNAs), such as small interfering RNAs (siRNAs), long noncoding RNAs (lncRNAs), microRNAs, and circular RNAs, regulate cellular function. Furthermore, ncRNAs are known to influence gene activity by modulating Nrf2 expression [196] and cellular responses to a redox state (Table 4) [197]. Recent research has specifically demonstrated that lncRNAs are capable of inhibiting Nrf2 expression through epigenetic mechanisms; this inhibition facilitates inflammasome activation, which, as observed for example in murine and microglial models of Parkinson’s disease (PD), triggers the neuroinflammatory cascade [198]. An adequate diet, characterized by a sufficient intake of fruits and vegetables, is essential for meeting nutritional needs and improving immune and antioxidant resilience against diseases associated with inflammation and oxidative stress. Recent studies reported that sulforaphane supplementation promoted Nrf2 pathway activation, which, in turn, mitigated and reversed the high-glucose-induced transcriptional and epigenetic alterations in vitro [199]. Likewise, other experimental studies have demonstrated that specific nutrients act as powerful antioxidants, both through direct scavenging of ROS and by increasing the expression of cellular antioxidant enzymes, inhibiting cellular damage induced by excessive oxidative stress [200,201]. Several studies report that oxidative stress is a determining factor in promoting various acute and chronic metabolic diseases, including diabetes, which subsequently leads to kidney damage. Some bioactive compounds can influence the *Nfe2l2* gene, effectively acting as modulators through epigenetic mechanisms [186], such as DNA methylation, histone modifications, and miRNA alterations. Given its protective role, the Nrf2 signaling network is considered a therapeutic target of great interest against metabolic diseases mediated by oxidative stress, including diabetes. These naturally occurring dietary compounds are capable of modulating the Keap1-Nrf2 pathway. This review examines in detail the involvement of the Nrf2 signaling pathway in chronic metabolic diseases, with particular reference to diabetes mellitus. It is well-documented that the administration of ursolic acid and tanshinone causes epigenetic modulation of the *Nfe2l2* gene. The identification of bioactive nutrients as modulators of the Nrf2 pathway represents a particularly promising area of research, as these natural compounds not only exhibit significant anti-inflammatory and antioxidant properties but also show documented evidence of their ability to regulate the expression of *Nfe2l2* through epigenetic mechanisms [202]. In this context, an emerging line of research explores the potential of bioactive nutrients as targeted effectors of the Nrf2 signaling cascade. It is increasingly evident that these natural compounds not only offer notable anti-inflammatory and antioxidant effects, but are also capable of influencing the *Nfe2l2* gene via epigenetic modifications [203]. These characteristics derive primarily from the ability of natural substances to modulate the Nrf2 signaling pathway, enhancing the body’s anti-inflammatory, antioxidant, and anti-apoptotic protective mechanisms [204,205]. Increased understanding of the molecular processes and regulation of the Nfe2l2 gene offers new opportunities for the prevention and treatment of various diseases. Furthermore, because epigenetic changes are not permanent, modulating these mechanisms is an extremely promising approach, especially using bioactive food components capable of acting at various stages, such as transcriptional, post-transcriptional, and post-translational [206]. Studies on the modulation of the human epigenome and the Nrf2 pathway by bioactive nutrients are attracting increasing interest. Although key points in the complex Nrf2 signaling network are now known, and molecules such as ursolic acid [201] and tanshinone [202] influence the epigenetics of Nrf2, many aspects remain to be clarified. Tanshinone is well known for its ability to enhance Nrf2 transcription at the promoter level [202]. Models of rifampicin-induced hepatotoxicity, both in in vitro and in vivo models, have demonstrated the induction of Nrf2 expression through the activation of epigenetic processes. However, no significant changes in DNMTs were detected. However, elevated production of DNA demethylases was found in human hepatocytes. Other research in in vitro cellular models has reported a reduction in methyltransferase enzymes, including Hdac1, Hdac3, and Hdac8 [207]. Furthermore, tanshinone IIA causes demethylation in the promoter of the *Nfe2l2* gene, thanks to the presence of the TET2 enzyme [202]. Numerous studies have investigated the role of ursolic acid in the epigenetic regulation of Nrf2 [201]. In vitro studies have shown that ursolic acid can stimulate elevated expression of a protein methyltransferase, such as SETD7. Other research has confirmed the relevance of Nrf2 protein methylation by SETD7 in gene silencing [208]. Other experiments have shown that ursolic acid activates Nrf2 through demethylation of the *Nfe2l2* gene in the promoter area, a mechanism that leads to a reduction in DNMT and HDAC enzymes [201]. A more detailed understanding of these molecular processes that regulate Nrf2 could provide new targets and strategies for the treatment of related diseases. Elucidating the regulatory mechanisms of Nrf2 and developing new targeted therapies requires identifying the interactions between its different alterations in the pathophysiological state. However, challenges remain, such as the complexity of interactions between genes, nutrients, and environmental pollutants, the need for basic and translational research, long-term human studies, and the development of precise tools for personalizing nutritional interventions to integrate these advancements into clinical practice.

## 8. Gene–Environment Interplay Between MNPs and *Nfe2l2*: Role of Nutrigenomics

Nutrigenomics examines the intricate interaction between nutrients and genes, focusing on how dietary signals influence gene expression, DNA structure, and metabolic processes to potentially prevent or manage chronic diseases like diabetes and its complications [209,210]. This multi-omics methodology, which combines genomics and redoxomics, analyzes the molecular processes underlying individual differences in response to dietary treatments, aiming to optimize metabolic health. From this new perspective, the approach highlights how tailored DNA-based dietary strategies, in particular polyphenol-enriched diets, can be aligned with specific genetic variants (*Nfe2l2* gene) and metabolic phenotypes (e.g., insulin resistance) to improve nutritional outcomes in a clinical setting [211]. Generally, genetic variations in the *Nfe2l2* gene and epigenetic modifications influence individual susceptibility to MNP-induced toxicity. MNPs directly disrupt Keap1-Nrf2 binding and epigenetic mechanisms primarily by triggering heavy ROS production, causing oxidative stress that releases Nrf2 from Keap1 [212]. Indeed, long-term exposure and high doses of MNPs inhibit the antioxidant Nrf2 pathway while activating inflammation, resulting in reduced intracellular defense systems and stress resilience genes, including *SOD*, *CAT*, and *GSH-Px* [213], and increase oxidative stress markers, such as MAD [212]. Moreover, current evidence has shown that PS-MPs and 17α-methyltestosterone induced a marked overexpression of pro-inflammatory genes, such as *TNF-α* and *IL-1β*, while simultaneously reducing the activity of cytoprotective genes such as *GPx4b*, *Nrf2*, and *HO-1*. This translates into an exacerbation of oxidative stress and inflammation through liver–brain communication in the zebrafish model [214]. Of note, MNPs leach toxic additives like phthalates, bisphenol A (BPA), and per- and polyfluoroalkyl substances (PFAS) that induce oxidative stress, which, in turn, affects the expression of genes involved in epigenetic regulation, causing genotoxicity, ultimately leading to abnormal gene silencing or overactivation [215,216]. In line with this notion, MPs and BPA co-exposure markedly inhibited the *Nfe2l2* gene, leading to the downregulation of antioxidant genes (*SOD1*, *CAT*, *GPx*), and upregulation of *Keap1* expression, increased hepatic ROS and MAD [217]. In aquatic organisms, exposure to low doses of 4.5 µg/L and 18 µg/L of polylactic acid (PLA) MPs caused intestinal mitochondrial dysfunction, significantly increasing mitochondrial ROS and markedly decreasing the expression of the oxidative stress genes *Gclc* and *Gpx1* via inhibition of the sestrin2/Nrf2 pathway in a dose-dependent manner [218]. These pathological mechanisms are often dose-dependent and can result in significant epigenetic dysregulation at the gene level, as they interfere with the expression of key miRNAs and the methylation process of critical genes involved in β-cell metabolism and glucose homeostasis, altering metabolic pathways [216]. Emerging studies show that food nutrients display multiple preventive and pharmacological activities against MNP damage and toxicity [219,220,221]. Notably, didymin is a dietary flavanone that ameliorated PS-NPs intoxication by enhancing the expression of *Nfe2l2* and antioxidant genes (*CAT*, *SOD*, *GPx*, *GSR*, *GST*, and *HO-1*) and reducing the expression of *keap1* and inflammatory genes (*IL-1β*, *TNF-α*, *IL-6*, *NF-κB*, and *COX-2*) in albino rats [219]. Nano-selenium mitigated PS-MPs-induced oxidative stress and intestinal microbiota changes through restoring the activities of the antioxidant *Nfe2l2* gene and related enzymes. Furthermore, nano-selenium supplementation lowered the transcriptional level of the immune-related genes (Toll-like receptor 4 (TLR4) and myeloid differentiation factor 88 (MyD88)), inflammation-related genes (major histocompatibility complex class II (MHC-II) and interleukin 8 (IL-8)) after PS-MPs exposure [209]. Mechanistically, melatonin (1 μM) activated the Nrf2-isl2a (ISL LIM homeobox 2a) axis to antagonize the side effects of doses of 0.5 and 25 mg/L of MPs [221]. Overall, preclinical data underscore the importance of limiting MP exposure to mitigate potential health hazards. Specifically, exposure to MNPs alters the *Nfe2l2* gene and the related antioxidant signaling, exacerbating oxidative stress and its pathological consequences. Nutrients interact with redox-dependent antioxidant genes, which are crucial for preventing or blocking MNP-induced genotoxicity, oxidative damage, and inflammation. Currently, there are no clinical studies evaluating the short- and long-term toxic effects of MNPs in humans. Further investigations are required to translate these promising findings into clinical practice, with the ultimate goal of promoting novel nutritional interventions for public health.

## 9. Advanced 3D Models for Studying Novel Nutritional Interventions in Diabetes

Three-dimensional models are instrumental in charting the gene expression and mutation patterns of the Nrf2 in several diseases, providing key information for understanding disease development, predicting progression, and identifying novel targets for nutritional interventions. Three-dimensional (3D) cell culture systems, particularly spheroids, have emerged as advanced in vitro models that better replicate the complex architecture, cell–cell interactions, and microenvironmental gradients found in native tissues compared to traditional two-dimensional (2D) cultures [222,223]. These models offer enhanced physiological relevance for investigating cellular behavior, drug responses, and disease mechanisms across a broad spectrum of biomedical fields, including oncology, tissue engineering, and metabolic disorders. Spheroids enable the study of cellular responses in conditions that closely mimic in vivo nutrient, oxygen, and signaling gradients, thereby providing critical insights into phenomena such as hypoxia, metabolic stress, insulin secretion, and extracellular matrix remodeling [224,225]. Consequently, 3D spheroids are increasingly employed to evaluate the efficacy and mechanisms of bioactive compounds, including polyphenols, which have shown protective effects due to their antioxidant and anti-inflammatory properties in metabolic diseases [226]. Specifically, eriocitrin and hesperidin, the most abundant flavanones in *Citrus lumia* peel extracts, at doses of 25 and 50 μg/mL, decreased intracellular lipids in both HepG2 cells and 3D spheroids [226]. This chapter focuses on the application of 3D models (spheroids and organoids) to assess the benefits of polyphenols in various pathological contexts, particularly in diabetes. We will review key studies demonstrating how 3D cultures enhance the understanding of polyphenol-mediated cytoprotection and metabolic modulation, highlighting the translational relevance of these findings for preventive and regenerative medicine in metabolic disorders, particularly in the management and therapy of diabetes (Table 5). Interestingly, 3D cultures have emerged as powerful tools to investigate oxidative stress-related cell death mechanisms, including apoptosis/pyroptosis, and to explore therapeutic strategies targeting the Nrf2 pathway and related redox resilience proteins. In diabetes research, 3D models have also shown promise. Subramaniam et al. developed bioprinted liver discoids with stable metabolic activity and high expression of genes involved in drug metabolism, outperforming conventional spheroid models [227]. The study by Kozyra et al. highlighted how 3D human liver spheroids allow us to understand the mechanisms underlying steatosis and IR. These three-dimensional models are indeed able to reproduce the pathological condition of hepatic steatosis, characterized by excessive diglyceride and triglyceride accumulation in the liver. This response is induced through activation of the FOXO1 pathway [228]. Moreover, a recent study performed by Morisseau et al. investigated in spheroid 3D islet-like cells the pancreatic responses to a diabetogenic environment. The authors demonstrated that spheroid 3D pancreatic β-like cells from iPSCs exposed to saturated free fatty acids, in particular palmitic acid (0.5 mM), for 72 h increased lipid and carbohydrate metabolism (HMGSC2, LDHA, and GLUT3), fibrin metabolism (FGG, FGB), apoptosis (CASP7), and reduced the activity of protective transcription factors and pathways, including Nrf1, hippo, FOXO, SP3, and TCF4. These mechanisms were associated with a defect of insulin secretion and death [229]. Importantly, high glucose and AGEs persistently elevated expression of DNMT1, but not DNMT3A and DNMT3B, despite glucose normalization. Mycophenolate mofetil, a pharmacological inhibitor of IMPDH2, decreased sustained levels of DNMT1 expression and impeded sprout formation in 3D endothelial cultures induced by transient hyperglycemic conditions during epigenetic and metabolic reprogramming, with clinical relevance to the pathogenesis of diabetic complications [230]. Within hepatocyte spheroids, high doses of insulin promoted the expression of genes encoding key enzymes for gluconeogenesis (ChREBP) and de novo lipogenesis (FASN, FOXO1, and SREBF1) [231]. These findings confirm that, under physiological conditions, glucose uptake is closely linked to insulin action. On the contrary, in insulin-resistant steatotic hepatocytes, this regulation is broken, making the entry of sugar dependent almost exclusively on glucose levels in the extracellular space [231]. An interesting study conducted by Campo et al. demonstrated the feasibility of vascularized iβ spheroids using decellularized lung scaffolds and hiPSC-derived β cells. Indeed, vascularized iβ spheroids effectively preserved β cell mass and physiologic insulin release via targeting miR-375 in vitro [232]. In addition, the same study also revealed that vascularized iβ spheroids transplanted in diabetic mouse models restored glycemic control [232]. Compelling studies suggested that food nutrients—particularly polyphenols, flavonoids, and terpenoids—act as potent antioxidant, anti-inflammatory, lipid-lowering, and insulin-signaling agents. Accordingly, a recent report by Chu and coworkers demonstrated that fucoidan, often used as a food supplement, ameliorated free fatty acid-induced lipid accumulation and oxidative stress by activating the PI3K/AKT/Nrf2 signaling pathway and inhibiting NF-κB-mediated inflammation in both spheroids and the HepG2 cells model of non-alcoholic fatty liver disease [233]. Together, these studies underscore the relevance of 3D spheroid systems in dissecting the molecular pathways of apoptosis/pyroptosis and oxidative stress, highlighting the Nrf2 axis as a key regulatory node and potential therapeutic target in both diabetes and complications. The transition from traditional 2D cell cultures to 3D models has significantly improved the biological relevance of in vitro systems for studying complex pathologies and testing novel nutritional interventions in the clinical setting.

## 10. Potential Limitations of the Study

Currently, there are significant gaps in our understanding of the systemic risk posed by micro- and nanoplastics, in addition to the lack of a multidisciplinary perspective. These limitations stem primarily from the technical difficulty in detecting MNPs and are attributable to the lack of standardized protocols for identifying MNPs in tissues and body fluids. Control systems to avoid contamination and facilitate comparisons between studies have not yet been established, allowing for a more realistic and comparable assessment of daily human exposure, given the heterogeneous nature of MNPs. There is still no standardization of animal models for dose-response studies on MNPs, to assess and compare the toxicity and mechanisms of action of different types and sizes of MNP particles, and there are still few long-term epidemiological studies evaluating the chronic effects of MNPs on humans. Today, addressing MNPs requires interdisciplinary collaboration and scientific and technological innovation. Strengthening communication between researchers is essential to developing sustainable solutions. The poor bioavailability of polyphenols, often combined with low aqueous solubility and rapid metabolism, significantly limits their antioxidant and anti-inflammatory efficacy. To overcome these challenges, the use of innovative nutritional strategies, including nanodelivery systems such as nanoencapsulation, aimed at protecting these active compounds and improving their absorption, could prove effective in chronic conditions such as diabetes.

## 11. Conclusions

The impact of MNPs on human health is an emerging field of research, and significant attention is currently being directed toward functional food nutrients that can counteract their negative effects on human health. The environmental invasion of such particles leads to food contamination. Through the diet, they are deposited in organic tissues, including the liver and immune system. The accumulation is related to the phenomena of cytotoxicity, inflammation, and genetic alterations, factors that favor the onset of chronic pathologies. Plant-derived compounds act in a dose-dependent manner. They modulate the *Nfe2l2* gene and stimulate the production of stress-resilient proteins, as well as antioxidant and detoxifying enzymes. This cellular defense mechanism limits the permanence of MNPs in the body. Consequently, oxidative stress and pyroptosis are reduced, thus preventing the progression of diabetes. The review examined selective functional nutrients, including baicalin, cynarin, tanshinones, verbascoside, and ursolic acid. While verbascoside and ursolic acid preserve the integrity of the intestinal barrier by limiting particle absorption, diosmin and tanshinone protect the vascular system from systemic toxicity. Finally, cynarin and baicalein enhance endogenous enzymatic defenses, targeting Nrf2, in metabolically active organs, counteracting IR and the organ damage typical of diabetic disorders aggravated by exposure to MNPs. The coherence of this approach lies in the role of Nrf2 as a molecular bridge between MNP-induced oxidative stress and metabolic resilience. The *Nfe2l2* gene/Nrf2 signaling pathway is subject to epigenetic regulation and significantly impacts cellular resilience. Furthermore, it is possible that some of their metabolites can activate the *Nfe2l2*/Nrf2 pathway systemically. Identifying specific food nutrients capable of activating at the minimum dose, the Nrf2 axis by reversing hypermethylated states in the CpG islands of the *Nfe2l2* gene, via the inhibition of DNMT and HDAC, offers a promising personalized approach in disease prevention and management. In summary, this study supports a shift toward sustainable personalized nutritional strategies based on bioactive nutrients, also known as “nutrigenetic and nutriepigenetic modulators,” i.e., supplements with high synergistic potential as adjuvants to traditional therapies in reversing genetic/epigenetic variations in the *Nfe2l2* gene to prevent or restore the risk of MNP-induced DNA damage and related chronic diseases such as diabetes in humans. This review is the first to interconnect these elements by outlining specific epigenetic signals induced by MNPs and the role of dietary nutrients in mitigating and/or counteracting cellular damage and MNP toxicity in chronic diseases.

## 12. Future Perspectives

Currently, there is an urgent need to shift from acute, high-dose studies to long-term, low-dose exposure models, and to better understand the long-term effects of MNPs. Translation from in vitro models to clinical practice requires a deeper understanding of optimal doses to avoid adverse effects or unwanted drug interactions, especially in vulnerable individuals such as diabetic patients. The rapid metabolization of molecules like baicalein limits its systemic efficacy, despite excellent in vitro results. Potential risks are associated with chronic use at high doses. Optimal results obtained in cells are often not replicated in humans because the body eliminates nutrients too quickly. High doses of polyphenols can clash with medications that diabetic patients already take, such as metformin or insulin. There is still no approved “standard dosage” to counteract microplastic damage, as most data still comes from animal models. For baicalein, although Phase I studies have demonstrated good tolerability, chronic use at high doses requires caution, as it could lead to drug interactions. Ursolic acid, while protective at moderate doses, has been associated with potential endoplasmic reticulum stress in some studies when administered at supraphysiological concentrations. This suggests the need for long-term clinical trials: (i) to better understand MNP cellular and tissue accumulation, considering the toxic dose, size, distribution, retention, and excretion associated with metabolic dynamics after exposure; (ii) to evaluate sustained impacts on DNA methylation and the histone modifications pattern; (iii) to optimize the bioavailability and effective dosages of dietary nutrients; and (iv) to personalize nutritional interventions that integrate genetic and epigenetic profiling. The integration of multi-omics approaches, such as redoxomics, genomics, and epigenomics, could improve the understanding of the molecular toxicity of MNP pollutants and help identify potential individual biological biomarkers and interindividual metabolic susceptibility. Furthermore, the use of personalized nutrition targeting specific genes that can improve glycemic control, reduce MNP-induced systemic toxicity, and pro-inflammatory processes should be considered in future studies to manage diabetes. We hypothesize that a deeper understanding of the interactions between nutrients and the *Nfe2l2* gene could provide novel strategies to promote cellular resilience to stress, thereby limiting the potential detrimental effects of MNPs on human health. Advances in this area, particularly the implementation of cutting-edge methodologies such as 3D models, represent a promising option within integrative medicine, with significant potential to enhance clinical outcomes, promote metabolic health, and strengthen physiological cellular resilience in diabetes and the associated consequences.

## Figures and Tables

**Table 1:** Summary of potential effects of baicalein and cynarin on upregulated ↑ or ↓ downregulated molecular pathways involved in diabetes and its complications.

Food Nutrients	Molecular Mechanisms	Outcomes	Ref.
Baicalein	↑ *Nfe2l2* ↓ NF-κB ↓ iNOS, TGF-β1	Alleviates cytotoxic mechanisms and preserves renal function by anti-hyperglycemic, antioxidant, and anti-inflammatory effects in vitro and in vivo	[66,67,68,69]
	↑ AMPK, GLUT2 ↓ p38MAPK and STAT3	Enhances glucose utilization and glycolysis and hepatic glycogen content in insulin-resistant HepG2 cells and animal models	[70,71]
	↑ KEAP1-Nrf2 axis	Reduces mitochondrial oxidative stress and prevents both microvascular and macrovascular complications of diabetes	[72]
	↓ FAS, SREBP-1c ↑ PPAR-α	Decreases systemic inflammation, hyperglycemia, insulin resistance, and hyperlipidemia in HFD mice	[73]
	↓ AMPKα, hs-CRP, FcγR ↓ NF-κB	Protects renal function from inflammatory mediators, leading to DN progression	[74,75]
	SphK1/S1P/NF-κB	Restores glucose and lipid metabolism and counteracts oxidative stress and inflammation in db/db mice and HK-2 cells	[76,77]
	↑ Nrf2, HO-1, NQO-1 ↑ GSH-PX, SOD, and CAT ↓ MDA, IL-1β, IL-6, MCP-1 and TNF-α ↓ MAPK	Reduces proteinuria, renal cell apoptosis, inflammation, and oxidative stress in animal experiments	[78]
	↓ PI3K/Akt/mTOR	Reduces creatinine, blood urea nitrogen, and albuminuria and improves renal histology in a murine model in a dose-dependent manner	[79]
	↓ TLR4/NF-κB	Attenuates renal fibrosis both in mice with streptozotocin-induced DN and in high glucose-treated HK-2 cells	[80]
	↑ SOD, GSH-PX ↓ NF-κB, VEGF	Reduces urinary markers (microalbuminuria, urinary β2-microglobulin, urinary albumin excretion rate) and inflammatory biomarkers and enhances antioxidant enzyme activities in DN patients	[81,82]
	↓ TRPV1, p38 MAPK ↓ P2Y12	Reduces neuronal damage leading to the development of neuropathic pain in a murine model dose-dependently	[67,83,84]
Cynarin and caffeic, ferulic acid, chlorogenic acid	↓ AGEs ↑ GPx, SOD	Protects plasma proteins and lipids against nitration and oxidation, increases the non-enzymatic antioxidant capacity of plasma in vivo	[85,86,87]
Artichoke water extract	↑ IRS1/PI3K/AKT/FoxO1 ↑ GSK3β	Inhibits endoplasmic reticulum stress and insulin resistance in HepG2 cells induced by palmitate dose-dependently	[88]
Cynarin	↑ CAT, SOD, GSH	Decreases blood glucose, total cholesterol, triglycerides, and LDL-C in diabetic rats	[89,90,91]
	↑ SOD, GPx	Reduces oxidative stress and inflammation, contributing to nerve cell damage associated with diabetes in vivo	[92,93]

Nfe2l2—nuclear factor erythroid-derived 2-like 2; NF-κB—nuclear factor kappa-light-chain-enhancer of activated B cells; iNOS—inducible nitric oxide synthase; TGF-β1—transforming growth factor beta 1; AMPK—5′AMP-activated protein kinase; GLUT2—glucose transporter 2; p38MAPK—p38 mitogen-activated protein kinases; STAT3—signal transducer and activator of transcription 3; KEAP1—Kelch-like ECH-associated protein 1; Nrf2—nuclear factor erythroid 2-related factor 2; FAS—fatty acid synthase; SREBP-1c—sterol regulatory element-binding protein 1; PPAR-α—peroxisome proliferator-activated receptor alpha; AMPKα—5′AMP-activated protein kinase alpha; hs-CRP—high-sensitivity C-reactive protein; FcγR—Fc gamma receptor; SphK1—sphingosine kinase 1; S1P—sphingosine-1-phosphate; HO-1—heme oxygenase-1; NQO-1—NAD(P)H quinone dehydrogenase 1; GSH-PX—glutathione peroxidase; SOD—superoxide dismutase; CAT—catalase; MDA—malondialdehyde; IL-1β—interleukin-1 beta; IL-6—interleukin-6; MCP-1—monocyte chemoattractant protein-1; TNF-α—tumor necrosis factor alpha; MAPK—mitogen-activated protein kinase; PI3K—phosphoinositide 3-kinase; Akt—protein kinase B; mTOR—mammalian target of rapamycin; TLR4—Toll-like receptor 4; VEGF; vascular endothelial growth factor; TRPV1—transient receptor potential vanilloid type 1; P2Y12—purinergic P2Y12 receptor; GSH—glutathione; GPx—glutathione peroxidase; IRS1—insulin receptor substrate 1; FoxO1—forkhead box protein O1; GSK3β—glycogen synthase kinase-3 beta.

**Table 2:** Summary of potential effects of diosmin and tanshinone on upregulated ↑ or ↓ downregulated molecular pathways involved in diabetes and its complications.

Food Nutrients	Molecular Mechanisms	Outcomes	Ref.
Diosmin	↓ aldose reductase, α-glucosidase, α-amylase	Inhibits the enzymes involved in diabetes and its complications more than the standard drugs in vitro	[94,95]
	↑ alanine aminotransferase, aspartate transaminase, and alkaline phosphatase	Ameliorates glucose intolerance and the serum markers of liver function, as well as decreases fasting blood sugar against sodium arsenite-induced toxicity in mice	[97]
	↑GRP78, CHOP ↑ SOD, CAT, MNSOD ↓ NOX2, MDA ↓ PI3K/AKT ↓ NLRP3	Reduces levels of inflammatory cytokines and increases antioxidant response in vivo	[98]
	↑ Sirt3 ↓ TNF-α, IL-6, IL-1β ↓ NF-κB p65	Counteracts renal fibrosis by reducing levels of inflammatory cytokines	[99]
	↑ SOD, CAT, GSH ↓ NF-kB ↓ MDA, NO	Reduces pro-inflammatory cytokines and enhances the activity of antioxidant enzymes	[100]
	↑ SOD, GSH ↓ MDA, NO	Inhibits oxidative stress markers and increases antioxidant enzymes in diabetic neuropathic rats	[101]
Diosmin and luteolin selenium nanoparticles	↑ CAT, SOD, GPx	Enhance insulin levels and antioxidant enzymes in the kidneys and livers of diabetic mice	[96]
Diosmin and hesperidin	↑ FGF21 ↓ Gal-3, MDA	Promote nerve regeneration accompanied by a reduction in perineural thickness, as confirmed by histopathological examinations in diabetic Wistar rats	[102]
	↑ NO/cGMP/PKG/KATP ↑ Iba-1, Olig2 ↓ IL-1β, IL-33/St2	Improves CCI-induced mechanical and thermal hyperalgesia in mice	[103]
Diosmin plus hesperidin	↓ TGs, LDL	Reduces blood glucose and lipid profile from baseline in T2DM patients and associated neuropathy	[104]
Tanshinone I	↓ IL-6, TNF-α	Inhibits insulin resistance by reducing Ser307 phosphorylation IRS-1, alleviating T2DM syndrome in rats	[105,106,107]
Tanshinone IIA	↓ AKT1, VEGFA, NOS3, PPARG, MMP9, VCAM1	Alleviates T2DM and stimulates cellular stress response in molecular-docking studies	[108]
	↓ glucose	Inhibits blood glucose levels in fasting healthy Wistar rats	[109]
	↑ AMPK ↑ PI3K/AKT ↓ NF-κB, IL-6, IL-8, TNF-α, CRP	Improves insulin resistance and glucose and lipid metabolism, and reduces chronic inflammation in vivo	[110,111,112]
	↓ TGF-β1, NF-κB ↓ α-SMA	Reduces glucose-induced renal fibrosis by inhibiting extracellular matrix deposition and epithelial–mesenchymal transition	[113,114,115,116]
	↑ HO-1	Counteracts oxidative stress, thereby slowing the progression of DN	[117]
	↓ PI3K/Akt/mTOR	Prevents podocyte damage by promoting autophagy and reducing inflammation in db/db mice and in MPC5 cells under high glucose	[118]
	ELAVL1-ACSL4	in murine podocyte cells under hyperglycemic conditions	[120]
	↑ SOD ↓ PERK	Alleviates renal dysfunction and morphological alterations in streptozotocin-induced diabetic murine models	[121]
	↑ Nrf2, MRP2/4 ↓ NLRP3 ↓ TNF-α, IL-1β, IL-6, IL-18	Facilitates the clearance of toxic intermediate metabolites from the kidney in vitro and in vivo	[122,123,124]
	↑ Nrf2/ARE ↑ SOD and CAT ↑ IL-10 ↓ NF-κB, MDA	Alleviates diabetic neuropathic pain caused by oxidative stress and inflammation in experimental models	[125,126,127,128,129,130]
Tanshinone IIA and salvianolic acid B	↓ PI3K/Akt/NF-κB	Improve glucose and lipid disorders, liver and kidney damage, and block kidney inflammation in early DN rats	[119]

GRP78—78 kDa glucose-regulated protein; CHOP—C/EBP homologous protein; SOD—superoxide dismutase; CAT—catalase MNSOD—manganese-dependent superoxide dismutase; NOX2—NADPH oxidase 2; MDA—malondialdehyde; PI3K—phosphoinositide 3-kinase; Akt—protein kinase B; NLRP3—NLR family pyrin domain containing 3; Sirt3—sirtuin-3; TNF-α—tumor necrosis factor alpha; IL-6—interleukin-6; IL-1β—interleukin-1 beta; NF-κB p65—nuclear factor NF-kappa-B p65 subunit; GSH—glutathione; NO—nitric oxide; FGF21—fibroblast growth factor 21; Gal-3—galectin-3; cGMP—cyclic guanosine monophosphate; PKG—protein kinase G; KATP—ATP-sensitive potassium channel; Iba-1—ionized calcium-binding adapter molecule 1; Olig2—oligodendrocyte transcription factor; IL-33—interleukin 33; St2—suppression of tumorigenicity 2; TGs—triglycerides; LDL—low-density lipoprotein; AKT1—RAC-alpha serine/threonine-protein kinase; VEGFA—vascular endothelial growth factor A; NOS3—nitric oxide synthase 3; PPARG—peroxisome proliferator-activated receptor gamma; MMP9—matrix metalloproteinase-9; VCAM1—vascular cell adhesion molecule 1; CRP—C-reactive protein; TGF-β1—transforming growth factor beta 1; α-SMA—alpha smooth muscle actin; HO-1—heme oxygenase-1; mTOR—mammalian target of rapamycin; ELAVL1—ELAV-like RNA binding protein 1; ACSL4—acyl-CoA synthetase long chain family member 4; PERK—protein kinase RNA-like endoplasmic reticulum kinase; MRP2—multidrug resistance-associated protein 2; MRP4—multidrug resistance-associated protein 4; IL-18—interleukin-18; ARE—antioxidant response element; IL-10—interleukin 10; NF-κB—nuclear factor kappa-light-chain enhancer of activated B cells.

**Table 3:** Summary of potential effects of ursolic acid and verbascoside on upregulated ↑ or ↓ downregulated molecular pathways involved in diabetes and its complications.

Food Nutrients	Molecular Mechanisms	Outcomes	Ref.
Ursolic acid	↑ LKB1-AMPK1-FOXO3	Reverses hyperglycemia-induced hepatic inflammation	[135]
	↑ GSH-Px, SOD ↑ Pdx-1, Ins-1, Ngn-3 ↓ Traf-6, Mapk-8, Traf-4	Decreases oxidative stress to regenerate pancreatic β cells to secrete insulin	[136]
	↑ IRS-1/PI3K/Akt/GSK-3	Reduces ROS and promotes the revascularization and re-epithelization in diabetic animal models and in vitro	[140,141,142]
	↑ PPARγ/GLUT-4	Promotes antidiabetic action by insulin sensitization in vitro	[143]
	↓ IL-6, IL-18, caspase-1, NLRP3	Reduces proteinuria, immune cell infiltration, and histopathological damage in the renal tissue	[141]
	↓ NF-κB p65-MDM2-Notch1 ↓ TNF-α, MCP-1, IL-1β	Decreases blood glucose levels, HbA1C, creatinine, uric acid, blood urea nitrogen, AST, ALT, ALP, and AGE formation in the plasma and kidney	[147,148,149,150]
	↓ TNF-α, IL-1β, ↓ MDA, carbonylated proteins	Reduces mechanical and thermal hyperalgesia and counteracts neuropathic pain	[151]
	↑ PPARγ ↓ TRPV1	Alleviates symptoms of diabetic peripheral neuropathy by reducing inflammation	[152,153]
	↑ CREB-BDNF	Long-term administration attenuates Aβ42 and P-tau levels in C57BL/6 J mice	[154]
Ursolic acid and roseus ethanolic extract	↑ CAT, SOD, GPx, GSH	Increase antioxidant enzymes and reduce blood glucose levels after 28 days against streptozotocin-induced diabetes in rats	[133]
Ursolic acid lactone	↑ AMPK	Increases glucose uptake in insulin-resistant muscle cells and reduces TG content in hepatocytes and adipocytes	[138]
Ursolic acid and exercise training	↑ Sirt1/eNOS ↓ MDA	Decreases oxidative stress markers and increases antioxidant potential in diabetic rats	[139]
Ursolic acid and oleanolic acid	↓ α-glucosidases	Exhibits potent antihyperglycemic effects by reducing blood glucose levels and the postprandial peak after sucrose, in vivo, ex vivo, and in silico	[144]
Ursolic acid and catechin, epicatechin, kaempferol, metformin, quercetin	↑ GLUT4, AKT2, AMPKα1 and AMPKα2	Ameliorate hyperglycemia and insulin sensitivity in vivo	[145]
Verbascoside	↓ ROS ↓ NF-κB	Prevents oxidative stress and inflammation-induced damage in the islets of Langerhans by preserving β-cell function	[162]
	↑ PPARγ and GLUT4 ↓ α-glucosidase	Reduces blood glucose levels in mice	[143]
	↑ AMPK/NOX4/NF-κB	Promotes renoprotective effects in HK-2 cells and in molecular docking studies	[157]
	↓ NADPH/oxidase-TGF-β/Smad	Prevents glucose elevation in both human renal tubular cells and diabetic mice	[164]
	↓ PI3K/AKT/NF-κB	Promotes renoprotective effects	[166]
	↓ AKT/GSK-3β	Protects podocytes from apoptosis	[165]
	↓ AMPK/NOX4/NF-κB ↓ IL-6, TNF-α	Attenuates inflammation and fibrosis in diabetic murine models	[157,167]
	↑ AKT ↓ caspase 3	Improves the severity of MPTP-induced peripheral dopaminergic neuropathy in vivo	[171]

LKB1—liver kinase B1 AMPK1—5′AMP-activated protein kinase catalytic subunit α1; FOXO3—Forkhead box O3; GSH-PX—glutathione peroxidase; SOD—superoxide dismutase; Pdx-1—pancreatic and duodenal homeobox 1; Ins-1—insulin-1; Ngn-3—neurogenin-3; Traf-6—tumor necrosis factor receptor—associated factor 6; Mapk-8—mitogen-activated protein kinase 8; Traf-4—tumor necrosis factor receptor—associated factor 6; IRS-1—insulin receptor substrate 1; PI3K—phosphoinositide 3-kinase; Akt—protein kinase B; GSK-3—glycogen synthase kinase 3; PPARγ—peroxisome proliferator-activated receptor gamma; GLUT-4—glucose transporter type 4; IL-6—interleukin-6; IL-18—interleukin-18; NLRP3—NLR family pyrin domain containing 3; NF-κB p65—nuclear factor NF-kappa-B p65 subunit; MDM2—mouse double minute 2 homolog; Notch1—neurogenic locus notch homolog protein 1; TNF-α—tumor necrosis factor alpha; MCP-1—monocyte chemoattractant protein 1; IL-1β—interleukin-1 beta; MDA—malondialdehyde; CAT—catalase; SOD—superoxide Dismutase; GPx—glutathione peroxidase; GSH—glutathione; AMPK—5′AMP-activated protein kinase; Sirt1—sirtuin-1; eNOS—endothelial NOS; AKT2—RAC-beta serine/threonine-protein kinase; AMPKα1—5′-AMP-activated protein kinase catalytic subunit alpha-1; AMPKα2—5′-AMP-activated protein kinase catalytic subunit alpha-2; ROS—reactive oxygen species; NOX4—NADPH oxidase 4; NADPH oxidase—nicotinamide adenine dinucleotide phosphate oxidase; TGF-β—transforming growth factor beta; Smad—mothers against decapentaplegic homolog; GSK-3β—glycogen synthase kinase-3 beta; AKT—protein kinase B.

**Table 4:** Epigenetic changes and biological effects targeting the *Nfe2l2* gene.

Molecular and Epigenetic Targets	Epigenetic Mechanism	Physiological and Functional Outcomes	Ref.
Chromatin	Structural rearrangement	Dysfunctional *Nfe2l2* gene expression and cellular homeostatic imbalance	[193]
CpG sites (Nrf2 promoter)	Excessive methylation	Transcriptional silencing of Nrf2 and reduced redox defense systems	[194]
DNMT enzymes	Suppression by bioactive dietary compounds	Decreased Nrf2 promoter methylation and elevated Nrf2 protein	[195]
TET demethylases	DNA demethylation at the Nrf2 promoter locus	Enhanced Nrf2 transcription and oxidative stress resilience	[196]
Non-coding RNAs (si, ln, mi, circ)	Epigenetic fine-tuning of Nrf2 activity	Maintenance of the redox environment and disease progression	[197]
lncRNA	Epigenetic silencing of Nrf2	Inflammasome activation and neuroinflammation in PD models	[198]
Nrf2	chromatin accessibility memories	Reversed transient high glucose induced transcriptional and epigenetic memory in human endothelial cells	[199]

Nfe2l2—nuclear factor erythroid-derived 2-like 2; DNMT—DNA methyltransferase; TET—ten–eleven translocation methylcytosine dioxygenases; si—small interfering RNAs; ln—long non-coding RNAs; mi—microRNAs; circ—circular RNAs; lncRNA—long non-coding RNAs.

**Table 5:** Summary of recent applications of 3D models in diabetes and nutritional interventions. Molecular pathways upregulated ↑ or ↓ downregulated.

3D Model Technique	Targets	Therapeutic Application/Purpose	Ref.
LX2 spheroids	↓ Lipids and ↑ ATP	Reduce intracellular lipid accumulation and increase cellular energy balance after treatment of *Citrus lumia* extract in a dose-dependent manner	[226]
Hepatic 3D spheroids	↑ FOXO1 and ↓ GSK3β	Mimic the pathological condition of steatosis and insulin resistance	[227,228]
Islet 3D spheroids	↑ HMGSC2, LDHA GLUT3, FGG, FGB, ↑ CASP7, ↓ NRF1, FOXO, Hippo	Investigate the pancreatic-like response to diabetogenic environment after palmitic acid exposure, which promotes defect of insulin secretion and death	[229]
3D endothelial cultures	↓ DNMT1, IMPDH2	Identify metabolic and epigenomic signatures of metabolic memory after pharmacological intervention under hyperglycemic conditions	[230]
Hepatocyte spheroids	↑ ChREBP, FASN, FOXO1, and SREBF1	High insulin concentrations promoted the induction of genes encoding key enzymes of gluconeogenesis and de novo lipogenesis	[231]
Vascularized iβ spheroids	↓ miR-375	hiPSC-β cells preserve physiologic insulin release in vitro and restore glycemic function in vivo	[232]
Spheroids and HepG2 cells	↑ PI3K/AKT/Nrf2 ↓ NF-κB	Fucoidan reduces free fatty acid-induced lipid accumulation and oxidative stress	[233]

ATP—adenosine triphosphate; FOXO1—Forkhead box protein O1; GSK3β—glycogen synthase kinase-3 beta; HMGSC2—3-hydroxy-3-methylglutaryl-CoA synthase 2; LDHA—lactate dehydrogenase A; GLUT3—glucose transporter 3; FGG—fibrinogen gamma chain; FGB—fibrinogen beta chain; CASP7—caspase-7; NRF1—nuclear respiratory factor 1; DNMT1—DNA (cytosine-5)-methyltransferase 1; IMPDH2—inosine-5′-monophosphate dehydrogenase 2; ChREBP—carbohydrate-responsive element-binding protein; FASN: Fatty acid synthase; FOXO1—Forkhead box protein O1; SREBF1—sterol regulatory element-binding transcription factor 1; miR-375—microRNA 375; PI3K—phosphoinositide 3-kinase; AKT—protein kinase B; NF-κB—nuclear factor kappa-light-chain enhancer of activated B cells.

## Data Availability

No new data were created or analyzed in this study; references are provided for all sources. Data sharing is not applicable to this article.
